# Investigating the Mechanisms of *Lycii fructus* in Treating Nonalcoholic Fatty Liver Disease and Diabetes Comorbidity Through Network Pharmacology and Molecular Dynamics

**DOI:** 10.1002/fsn3.70256

**Published:** 2025-05-26

**Authors:** Peng Sun, Jiahui Song, Yang Liu, Xiujing Li, Yiming Zhang, Yuxing Zhou, Wei Gong

**Affiliations:** ^1^ Science and Technology Center Ningxia Medical University Yinchuan China; ^2^ Ningxia Hui Autonomous Region Institute of Medical Sciences Yinchuan China; ^3^ Public Health School Ningxia Medical University Yinchuan China; ^4^ School of Pharmacy Ningxia Medical University Yinchuan China; ^5^ Key Laboratory of Environmental Factors and Chronic Disease Control Ningxia Medical University Yinchuan China; ^6^ School of Medical Information and Engineering Ningxia Medical University Yinchuan China

**Keywords:** Comorbidity, *Lycii fructus*, Mendelian randomization, Molecular docking, Network pharmacology

## Abstract

Non‐alcoholic fatty liver disease (NAFLD) and diabetes mellitus (DM) are prevalent metabolic disorders that frequently coexist, yet their shared molecular mechanisms remain poorly understood, and current therapies often yield suboptimal outcomes. *Lycium barbarum* L. (*Lycii fructus*, LF), a traditional medicinal herb, has demonstrated clinical efficacy in treating both conditions, but its mechanism of action in comorbidity management remains unclear. Active LF compounds were identified via the TCMSP database, with potential targets predicted using Swiss Target Prediction and PharmMapper. Disease‐associated proteins for NAFLD and DM were curated from OMIM, GeneCards, DisGeNET, UniProt, DrugBank, and TTD. A protein–protein interaction (PPI) network was constructed from these targets, and GO and KEGG pathway analyses were performed using the DAVID platform. Key targets were further refined through network module analysis via Metascape. Drug‐likeness of bioactive compounds was assessed using SwissADME and ADMETlab 2.0. Molecular docking and dynamics simulations validated interactions between core targets and LF compounds. Mendelian randomization (MR) analysis tested causal relationships between core genes and disease phenotypes. We identified 58 shared therapeutic targets for NAFLD‐DM comorbidity, including HSP90AA1, ESR1, MMP9, EGFR, AKT1, and CASP3. GO analysis implicated LF in blood pressure regulation and glucose‐stimulated insulin secretion. KEGG pathways highlighted modulation of MAPK, PI3K‐Akt, FoxO, and mTOR signaling. 24‐methylenelanost‐8‐enol and cryptoxanthin monoepoxide emerged as core bioactive compounds with favorable drug‐likeness. Molecular docking confirmed strong binding of 24‐methylenelanost‐8‐enol to HSP90AA1 and cryptoxanthin monoepoxide to MMP9, further supported by dynamics simulations. MR analysis revealed a significant causal role for CASP3 in both NAFLD and DM, aligning with network pharmacology predictions. LF's therapeutic effects on NAFLD‐DM comorbidity likely arise from terpenoid and cryptoxanthin mediated modulation of apoptosis and inflammation pathway. This study identifies shared molecular networks, proposes candidate mechanisms for LF's efficacy, and provides a framework for targeting multifactorial metabolic diseases.

## Graphical abstract

Our study explores the mechanisms by which *Lycii fructus* (LF) treats the comorbidity of nonalcoholic fatty liver disease (NAFLD) and diabetes mellitus (DM). Through network pharmacology, key LF ingredients were identified, including terpenoids and cryptoxanthin monoepoxide, which modulate critical signaling pathways like MAPK and PI3K‐Akt by targeting proteins, such as HSP90AA1 and MMP9, offering potential therapeutic insights.

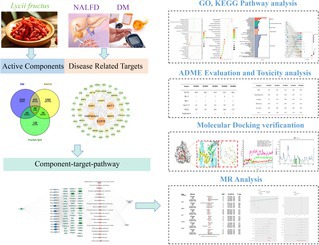

## 1 Introduction

Non‐alcoholic fatty liver disease (NAFLD), affecting about 25% of the global population, is a leading chronic liver disorder, with China reporting the highest prevalence and mortality rates in Asia (Younossi et al. 2023). NAFLD frequently coexists with metabolic comorbidities, particularly diabetes mellitus (DM), driven by shared risk factors, such as insulin resistance, dyslipidemia, and obesity (Mantovani et al. 2020; Kosmalski et al. 2022; Li et al. 2022). Notably, 56% of diabetic patients exhibit concurrent NAFLD (Younossi et al. 2019), amplifying risks of hepatic complications, cardiovascular disease, and extrahepatic malignancies (Qi et al. 2021; Wijarnpreecha et al. 2021). Mendelian randomization (MR) studies confirm a bidirectional causal relationship between NAFLD and DM, where each condition accelerates the progression of the other (Wang et al. 2023). DM exacerbates NAFLD‐driven liver fibrosis and doubles cirrhosis‐related mortality (Zoppini et al. 2014), whereas glycemic variability, reflected in HbA1c fluctuations, directly correlates with fibrosis severity (Alexopoulos et al. 2021). This intertwined pathogenesis, rooted in multifactorial mechanisms, limits the efficacy of single‐target therapies (Mantovani and Dalbeni 2021; Lee et al. 2022).

Traditional Chinese medicine (TCM), with its multicomponent, multitarget paradigm, offers a promising alternative. *Lycium barbarum* L. (*Lycii fructus*, LF), a widely used medicinal herb in China, exemplifies this approach. Rich in bioactive compounds, including polysaccharides (LBP), flavonoids (e.g., quercetin), and carotenoids, LF exhibits various pharmacological effects, such as immunomodulation (Ying and Hao 2023), antiaging (Gao et al. 2017), hypoglycemic (Su et al. 2024), lipid‐lowering (de Souza Zanchet et al. 2017), and antitumor activities (Xie et al. 2016; Manthey et al. 2017). Mechanistically, LBP modulates glucose metabolism via gut microbiota regulation (Li et al. 2023), whereas quercetin reduces DM risk (Li et al. 2020). Despite its therapeutic potential and established safety profile (Wang et al. 2019; Pai et al. 2013; Luo et al. 2004), LF's mechanisms in NAFLD‐DM comorbidity remain unclear.

To address this gap, we integrated network pharmacology and computational approaches. Network pharmacology, a systems biology‐based framework, identifies multi‐target interactions between herbal compounds and disease pathways (Zhao et al. 2023; Noor et al. 2023), whereas molecular docking predicts ligand‐receptor binding dynamics (Bai et al. 2023). Furthermore, MR, a genetic instrumental variable method, strengthens causal inference by mitigating confounding biases (Richmond and Davey Smith 2022), complementing network‐based predictions. This study aims to identify the active compounds of LF and their shared targets in NAFLD and DM, map enriched biological pathways through GO and KEGG analysis, and construct a component‐target‐pathway network to prioritize key bioactive compounds. We would also validate compound‐target binding stability using molecular dynamics simulations and apply MR to test causal relationships between core genes and disease phenotypes. By integrating these approaches, we aimed to elucidate LF's synergistic mechanisms in the comorbidity of NAFLD and DM, bridging traditional medicine with modern causal inference and systems pharmacology.

## 2 Methods

### 2.1 Identification of Bioactive Compounds and Targets in LF

The bioactive compounds in LF were retrieved from the Traditional Chinese Medicine Systems Pharmacology Database (TCMSP, Version 2.3; https://old.tcmsp‐e.com/tcmsp.php) (Ru et al. 2014). Screening criteria included oral bioavailability (OB) > 30% and drug‐likeness (DL) > 0.18 to prioritize pharmacologically active compounds. To validate the presence of TCMSP‐derived compounds in LF, PubMed, and Web of Science were systematically searched using keywords, such as *Lycii fructus*, *chemical constituents*, and *bioactive compounds*. The final validated compounds are listed in Table S1. Putative targets of the validated compounds were predicted using SwissTargetPrediction (http://swisstargetprediction.ch/) and PharmMapper (http://www.lilab‐ecust.cn/pharmmapper/).

### 2.2 Prediction of NAFLD and DM Targets

To determine potential targets associated with NAFLD and DM, a systematic search of databases was conducted using the keywords “Non‐alcoholic fatty liver disease” and “diabetes mellitus.” The databases queried include OMIM (https://www.omim.org/) (Amberger et al. 2015), GeneCards (https://www.genecards.org/), DisGeNET (https://www.disgenet.org/) (Piñero et al. 2020), TTD (http://db.idrblab.net/ttd/), UniProt (https://www.uniprot.org/) (UniProt Consortium 2019), and DrugBank (https://go.drugbank.com/).

### 2.3 Protein–Protein Interaction Network Analysis

The common targets between LF, NAFLD, and DM were identified, and the intersecting targets were uploaded to the STRING database (https://string‐db.org/) to construct a protein–protein interaction (PPI) network (Szklarczyk et al. 2023). The network was visualized using Cytoscape 3.9.1, with nodes arranged by degree. Nodes with higher degrees were represented by larger sizes, reflecting their greater importance within the network. This PPI network was constructed to elucidate the interactions between the overlapping target proteins associated with LF, NAFLD, and DM comorbidity, highlighting the key proteins involved in LF's therapeutic mechanisms. The species was set to *Homo sapiens* , and the confidence level was set at ≥ 0.7. To identify key nodes, network topological parameters, including degree, betweenness, and closeness, were analyzed using the CytoNCA plugin in Cytoscape (Assenov et al. 2008).

### 2.4 GO and KEGG Pathway Enrichment Analysis

To elucidate the mechanisms of LF in treating NAFLD and DM comorbidity, the intersected targets were subjected to Gene Ontology (GO) functional analysis and Kyoto Encyclopedia of Genes and Genomes (KEGG) pathway enrichment analysis. These analyses were conducted using DAVID 6.8 (https://david.ncifcrf.gov/) (Huang da et al. 2009). Next, we used KEGG Mapper to perform pathway analysis and collected protein data related to the highest degree of enrichment, focusing on key regulatory points and protein interactions. Finally, we visualized the highest degree of enrichment pathway using the KEGG website.

### 2.5 MCODE Analysis

MCODE analysis identifies protein relationships within molecular complexes and clusters them into gene clusters with similar functions. The key targets identified were imported into Metascape and analyzed using the KEGG pathway option (Bader and Hogue 2003). The parameters were set as follows: Min Overlap = 3, *p* value Cutoff ≥ 0.01, and Min Enrichment = 1.5. This enabled the identification of highly connected subnetworks in the PPI network.

### 2.6 Compounds‐Targets‐Pathway Network

To determine the main active components and core targets of LF and explore the interactions between targets and pathways, a compound‐target‐pathway network was constructed. This network was constructed by integrating the active components of LF, the overlapping targets of NAFLD and DM comorbidity, and the top 20 pathways from KEGG enrichment analysis. The network was also built using Cytoscape 3.9.1. A compound‐target‐pathway network was constructed, and network topology parameters were analyzed and sorted by degree value.

### 2.7 Bioactives and Drug‐Likeness Property of the Core Components of LF

SWISS ADME provides a broader assessment of bioavailability, considering not only oral bioavailability but also other ADME parameters, such as distribution, metabolism, excretion, Caco‐2 permeability, and blood–brain barrier penetration. SWISS ADME uses multiple computational models and physicochemical properties (e.g., log *p*, molecular weight, polar surface area) to predict bioavailability. The bioactive substances in LF were analyzed using Swiss ADME (http://www.swissadme.ch/) and then filtered according to Lipinski's rules to confirm physicochemical properties of “drug similarity,” which allows for a more comprehensive assessment of both ADME and drug‐like properties, providing a robust pharmacokinetic and toxicological evaluation to support drug screening and development.

### 2.8 Molecular Docking

Molecular docking was carried out to predict the binding affinities between the active compound and five core proteins identified from the PDB database (https://www.rcsb.org/). The proteins were optimized using Schrödinger's Protein Preparation Wizard, and receptor grids were generated based on ligand interactions. Docking was performed with Glide software in standard precision mode, and the binding interactions were further analyzed using Maestro 12.8.

### 2.9 Molecular Dynamics Simulations

Molecular dynamics (MD) simulations were performed using the Desmond module in Schrödinger software. The protein‐ligand complex was prepared by adding hydrogen atoms and removing water molecules to minimize the protein structure. The system was solvated using the TIP3P water model, and the OPLS3 force field was applied. Physiological conditions were simulated by adding sodium ions (Na^+^). The MD simulations were run using default parameters, and the results were analyzed with the simulation interactions diagram function. The root mean square deviation (RMSD) metric was employed to evaluate the stability and conformational changes of the protein‐ligand complex.

### 2.10 Mendelian Randomization Analysis

#### 2.10.1 Research Design of Mendelian Randomization

This study aimed to investigate the causal relationship between six key genes (EGFR, AKT1, ESR1, MMP9, HSP90AA1, CASP3) and DM, NAFLD using two‐sample MR analysis. By utilizing genetic variations as instrumental variables, MR analysis can infer causal relationships between exposures and outcomes, addressing confounding biases inherent in traditional observational studies and providing more reliable causal inferences. Additionally, various MR methods (e.g., weighted median analysis, IVW method) would be employed to evaluate the association and causal effects, providing robust evidence to further understand the biological mechanisms linking genes and diseases.

#### 2.10.2 Data Sources

The SNPs used in this study were derived from multiple published genome‐wide association studies (GWAS). All SNP data related to NAFLD and DM are publicly available. Specifically, SNP data for the six candidate genes (EGFR, AKT1, ESR1, MMP9, HSP90AA1, CASP3) as well as for DM and NAFLD were obtained from different GWAS datasets. The sample sizes and the number of SNPs for each dataset are summarized in Table 1.

**TABLE 1:** A summary of the data for MR analysis.

	Dataset	#SNPs in GWAS	Sample size	European	Year	Author
EGFR	prot‐a‐909	10,534,735	3301	European	2018	Sun BB
AKT1	eqtl‐a‐ENSG00000142208	20,441	30,721	European	2018	Vosa U
ESR1	eqtl‐a‐ENSG00000091831	19,600	26,609	European	2018	Vosa U
MMP9	eqtl‐a‐ENSG00000100985	18,885	31,684	European	2018	Vosa U
HSP90AA1	eqtl‐a‐ENSG00000080824	18,463	31,644	European	2018	Vosa U
CASP3	eqtl‐a‐ENSG00000164305	20,415	31,684	European	2018	Vosa U
T2DM	bbj‐a‐153	8,885,694	210,865	East Asian	2019	Ishigaki K
NAFLD	ebi‐a‐GCST90054782	9,097,254	377,998	European	2021	Fairfield CJ

#### 2.10.3 Instrumental Variable Selection

Instrumental variable (IV) selection in this study followed strict criteria. First, SNPs significantly associated with the exposure variables (EGFR, AKT1, ESR1, MMP9, HSP90AA1, CASP3) were selected, with a significance threshold of *p* < 1 × 10^−5^. To ensure the independence of the IVs, SNPs with strong linkage disequilibrium (LD) with other SNPs were excluded, setting criteria of *r* ^2^ < 0.01 and a distance threshold of 1000 kb. Additionally, the “mv_harmonise_data” function from the “TwoSampleMR” package was used to align effect alleles and effect sizes, ensuring consistency across SNPs. To further ensure the independence of the IVs, the “mv_lasso_feature_selection” function was employed to remove collinear variables. This process ensured that the selected SNPs were effective instrumental variables for subsequent MR analysis, enabling us to examine the causal relationship between the six key genes, DM and NAFLD.

#### 2.10.4 MR Analysis

The MR analysis in this study was conducted using “TwoSampleMR 0.6.8” and “MR‐PRESSO 1.0” packages. A variety of MR methods were used, including inverse variance weighting (IVW), Mendelian randomization‐Egger (MR‐Egger), weighted median (WM), simple model, and weighted model. The IVW method was used as the primary analysis method, with a *p* value threshold of < 0.05 indicating potential causality. To assess the robustness of the MR results, we performed several sensitivity analyses, including heterogeneity tests, horizontal pleiotropy tests, and one‐by‐one exclusion analyses. Cochran's *Q* statistic was used to evaluate heterogeneity in the IVW results, with a *p* value of < 0.05 indicating significant heterogeneity. MR‐Egger regression and MR‐PRESSO were employed to test for horizontal pleiotropy, with *p* < 0.05 suggesting the presence of pleiotropy. Additionally, the one‐by‐one exclusion method was used to remove individual SNPs and verify the consistency and robustness of the results. All analyses were performed using R 4.2.3.

## 3 Results

### 3.1 Prediction and Identification of Active Components and Targets of LF

From an initial pool of 45 active ingredients identified through TCMSP, 17 were retained after filtering for those with associated human target proteins (Table 2). By integrating data from multiple databases, 3346 target proteins were initially predicted. After eliminating duplicates, 266 unique targets were identified (Table S1).

**TABLE 2:** Detailed information of active compounds in LF.

No.	Mol ID	Molecule name	Structure	OB (%)	DL
1	MOL000953	Cyclolanostrol	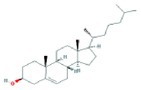	37.87	0.68
2	MOL001979	Lanosterol	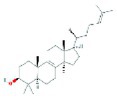	42.12	0.75
3	MOL005406	Atropine	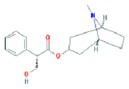	45.97	0.19
4	MOL008173	Daucosterol	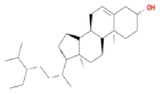	36.91	0.75
5	MOL009604	14b‐pregnane	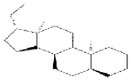	34.78	0.34
6	MOL009612	(24R)‐4alpha‐Methyl‐24‐ethylcholesta‐7,25‐dien‐3beta‐ylacetate	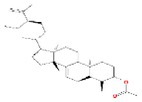	46.36	0.84
7	MOL009615	24‐Methylenecycloartan‐3beta,21‐diol	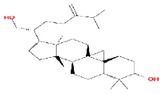	37.32	0.80
8	MOL009620	24‐methyl‐31‐norlanost‐9(11)‐enol	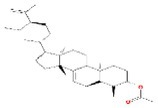	38.00	0.75
9	MOL009621	24‐methylenelanost‐8‐enol	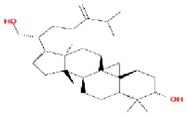	42.37	0.77
10	MOL009633	31‐norlanost‐9(11)‐enol	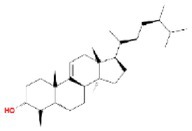	38.35	0.72
11	MOL009639	Lophenol	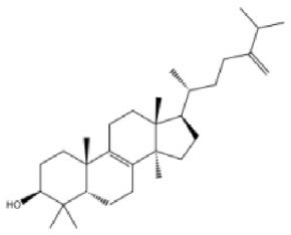	38.13	0.71
12	MOL009644	6‐Fluoroindole‐7‐Dehydrocholesterol	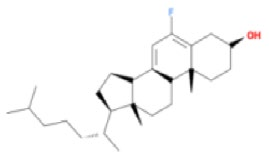	43.73	0.72
13	MOL009646	7‐O‐Methylluteolin‐6‐C‐beta‐glucoside_qt	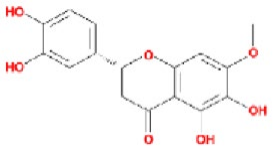	40.77	0.30
14	MOL009651	Cryptoxanthin monoepoxide	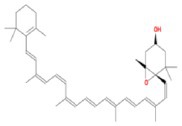	46.95	0.56
15	MOL009660	methyl (1R,4aS,7R,7aS)‐4a,7‐dihydroxy‐7‐methyl‐1‐[(2S,3R,4S,5S,6R)‐3,4,5‐trihydroxy‐6‐(hydroxymethyl)oxan‐2‐yl]oxy‐1,5,6,7a‐tetrahydrocyclopenta[d]pyran‐4‐carboxylate	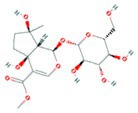	39.43	0.47
16	MOL009664	Physalin A	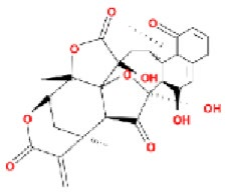	91.71	0.27
17	MOL009678	lanost‐8‐enol	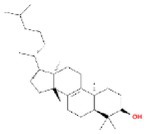	34.23	0.74

### 3.2 Prediction and Screening of NAFLD and DM Targets

For NAFLD, a total of 1961 targets were gathered from various databases, including 1342 from GeneCards, 528 from OMIM, 73 from DisGeNET, 9 from TTD, 5 from DrugBank, and 4 from UniProt. After duplicate removal, 1853 unique targets remained (Table S2). Similarly, 4669 DM‐related targets were identified, including 2416 from GeneCards, 1137 from DisGeNET, 789 from UniProt, 224 from OMIM, 100 from TTD, and 3 from DrugBank. After filtering duplicates, 4333 unique targets were obtained (Table S3).

### 3.3 Protein–Protein Interaction Network Analysis

**FIGURE 1:**
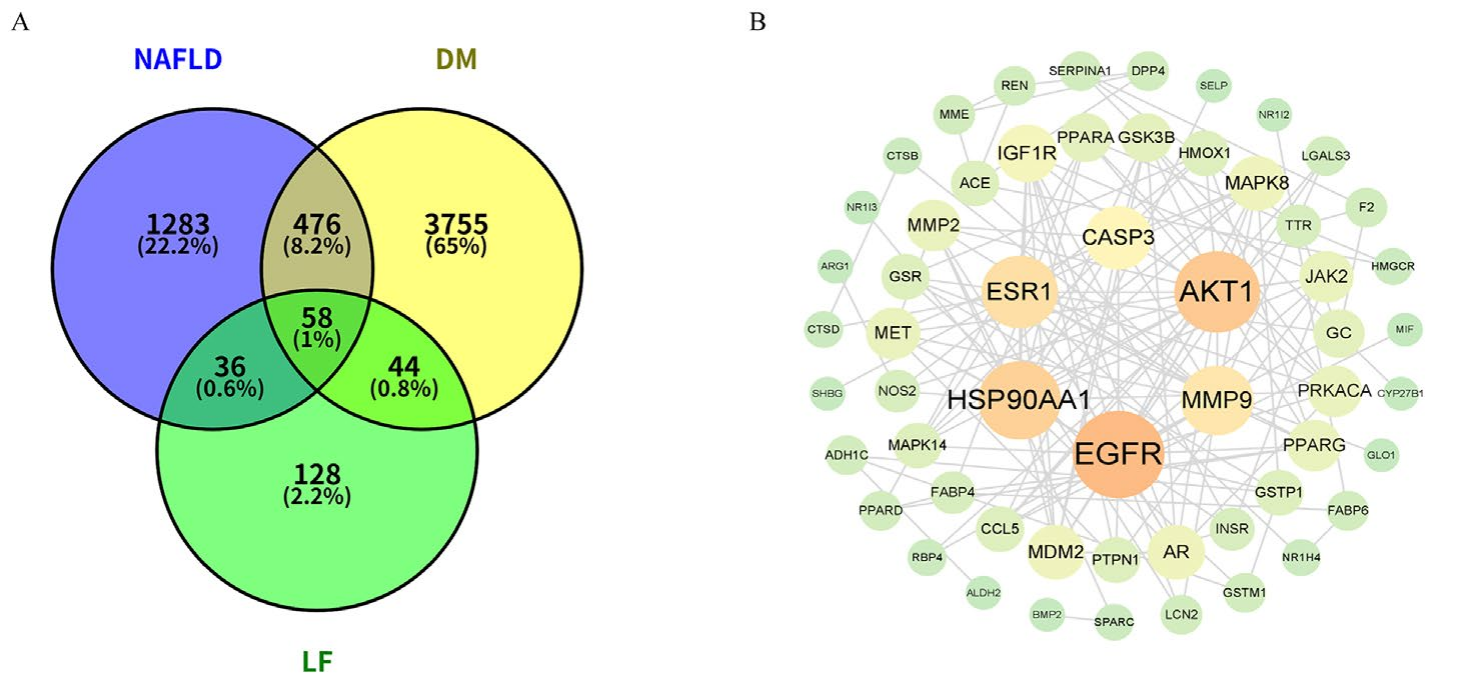
Intersection targets of LF in the treatment of NAFLD and DM comorbidity. (A) The Venn diagram. (B) PPI network of overlapping target proteins of LF, NAFLD, and DM comorbidity.

The targets of LF components were intersected with the targets of NAFLD and DM comorbidity. The resulting data were imported into VENNY, identifying 58 key targets of LF for the treatment of NAFLD and DM comorbidity (Figure 1A, Table S4). To elucidate the interactions between the overlapping target proteins of LF, NAFLD, and DM comorbidity, a PPI network was constructed using Cytoscape 3.9.1. In this network, nodes represent proteins, whereas edges denote interrelationships. This network, described in Table S5, consists of 56 nodes and 141 edges, which represent a specific subset of the entire network, focusing on key interactions and nodes for further analysis. Notably, key node proteins in the network include EGFR, AKT1, HSP90AA1, ESR1, MMP9, and CASP3 (Figure 1B).

### 3.4 GO Functional and KEGG Pathway Enrichment Analysis

**FIGURE 2:**
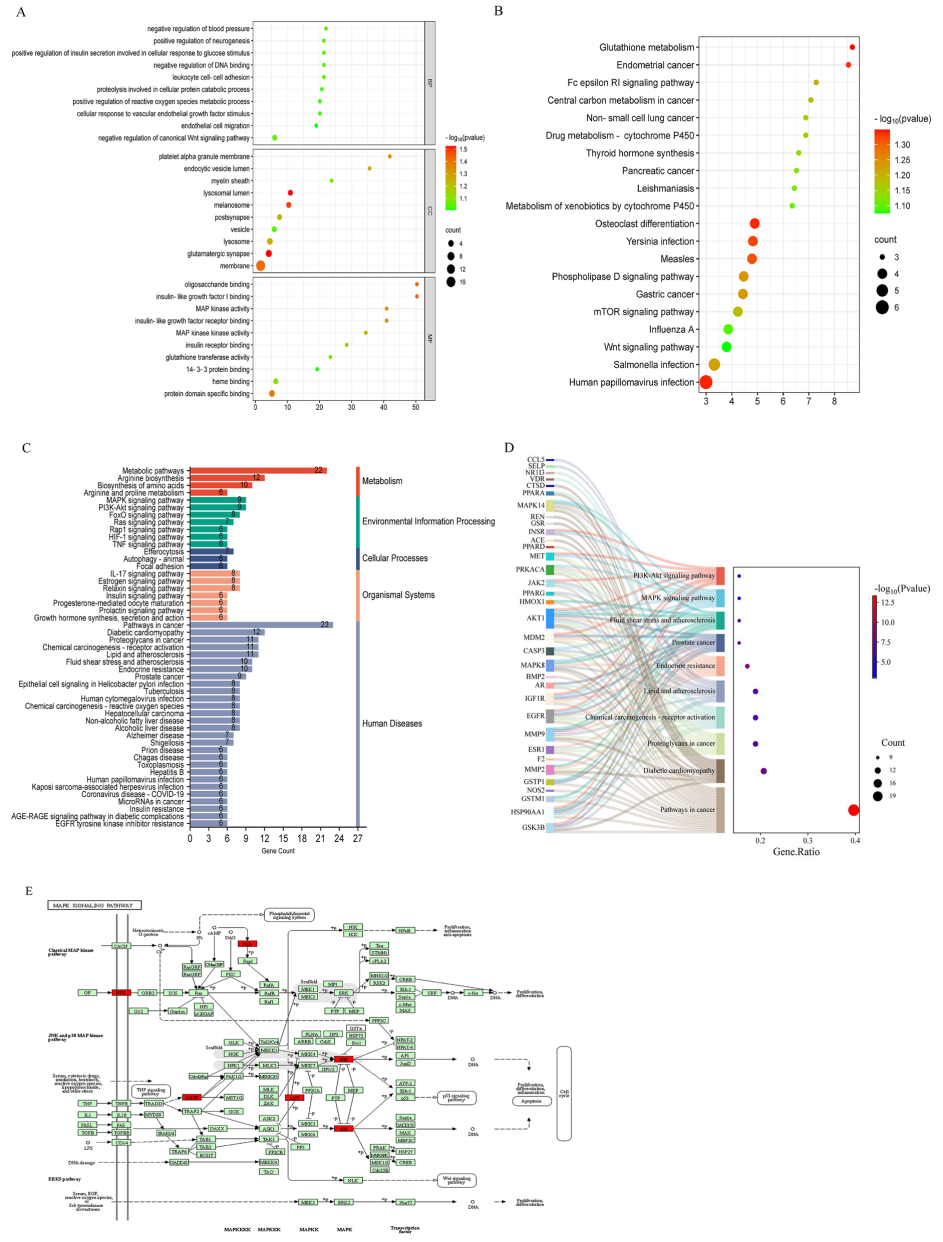
Key targets of LF for the treatment of NAFLD and DM comorbidity. (A) GO enrichment analysis. (B) KEGG pathway enrichment analysis. (C) Classification of KEGG pathway enrichment results. (D) The relationship between the first five key pathways in the enrichment pathway and related key genes. (E) Interactive analysis of the major MAPK signaling pathways with the red rectangle representing key targets.

The intersecting targets from LF and the NAFLD‐DM comorbidity targets were subjected to GO and KEGG pathway enrichment analysis using the DAVID database. A total of 345 GO terms were significantly enriched, comprising 247 biological processes (BPs), 36 cellular components (CCs), and 62 molecular functions (MFs) (Table S6). BPs were predominantly associated with endothelial cell migration, vascular endothelial growth factor regulation, reactive oxygen species metabolism, and positive regulation of insulin secretion. CCs were largely linked to the cell membrane, lysosomes, and vesicles, whereas MFs were involved in protein structure‐specific binding, glutathione transferase activity, insulin‐like growth factor binding, and oligosaccharide binding. The top 10 terms, filtered by a *p* value < 0.05, are displayed as bubble plots (Figure 2A). KEGG pathway enrichment analysis revealed that the active components of LF exert effects on NAFLD and DM comorbidity primarily through the Wnt, mTOR, and Phospholipase D signaling pathways (Figure 2B, Table S7).

Furthermore, analysis using the KEGG Mapper tool identified 306 enriched pathways: 64 related to metabolism, 29 to genetic information processing, 20 to environmental information processing, 81 to cellular processes, and 96 to organismal systems. These pathways were ranked by the number of enriched genes, and the top 20 pathways are illustrated in a bubble plot (Figure 2C). To further elucidate the mechanism of action of LF in treating NAFLD and DM comorbidity, the top five pathways, mTOR, MAPK, FoxO, PI3K‐Akt, and Phospholipase D signaling pathways, were selected based on the number of enriched genes and displayed in a network diagram (Figure 2D). Key proteins, such as EGFR, AKT1, INSR, INS, and IGF1R, which are strongly correlated with inflammation, stress, and metabolism, were highlighted. Among these, the MAPK signaling pathways showed the highest enrichment, suggesting their prominent role in the therapeutic effects of *LF* on NAFLD and DM comorbidity. Figure 2E visualizes the MAPK pathway using the KEGG website, with red‐marked regions indicating key interaction sites identified by PPI analysis. Other colors denote different signaling pathways and interaction types. The MAPK pathway plays a critical role in regulating cell proliferation, differentiation, apoptosis, and stress responses. Within the MAPK signaling pathway, an external signal (EGFR) binds to a cell surface receptor, leading to its activation. The activated receptor recruits aptamers and exchange factors, which subsequently activate small G proteins. These small G proteins then activate MAPK kinase (MAPKK), which in turn activates MAPK. This cascade further activates downstream inflammatory pathways, leading to the expression of proteins, such as CASP, JNK, AKT, and P38, ultimately triggering apoptosis.

### 3.5 MCODE Analysis of Intersection Targets of NAFLD and DM Comorbidity

**FIGURE 3:**
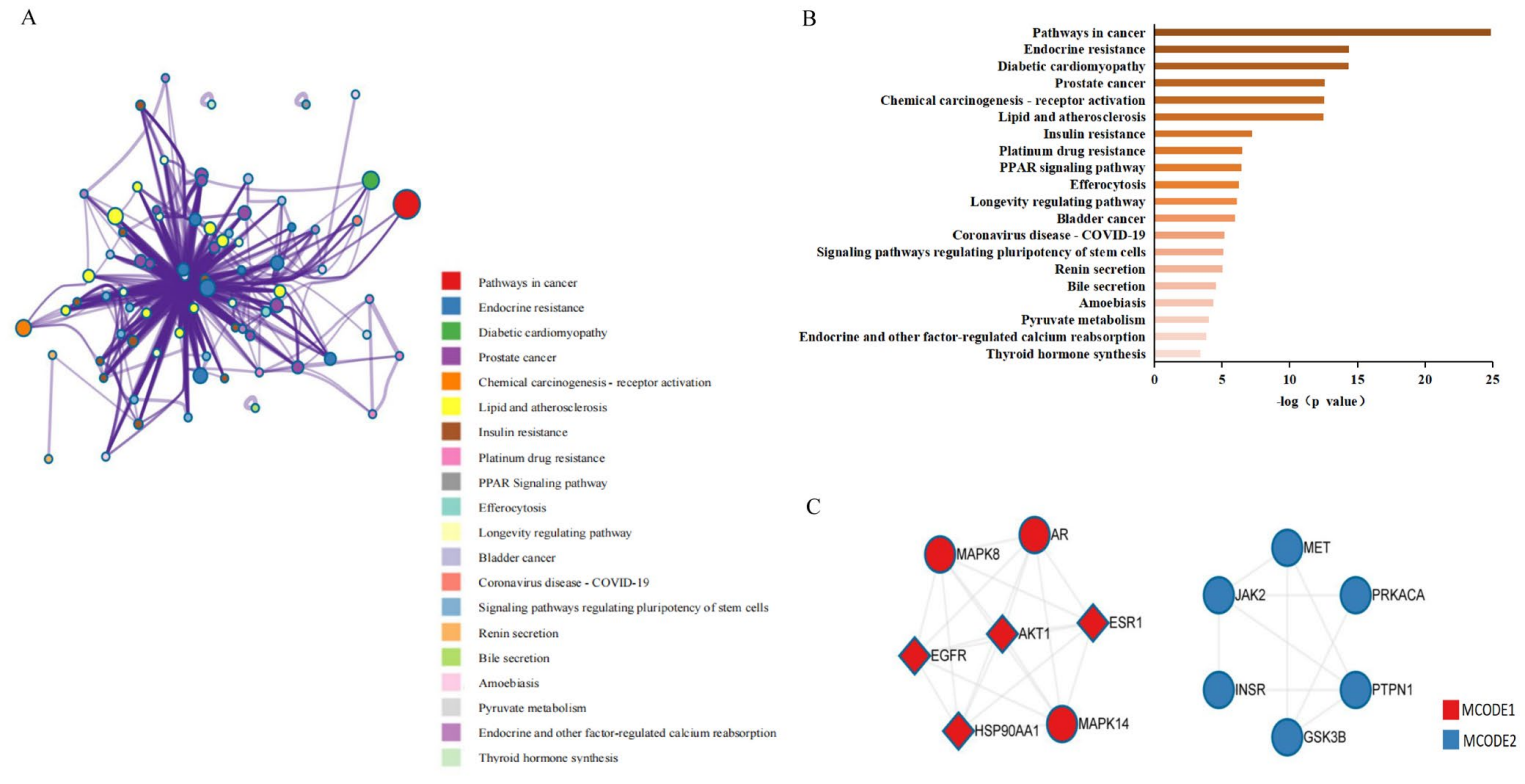
MCODE cluster analysis and protein function module of LF for NAFLD and DM comorbidity. (A) Sub network specific to the interaction. (B) Highly enriched terms of LF for NAFLD and DM. (C) Cluster analysis of LF for NAFLD and DM.

To further investigate the mechanisms by which LF regulates the NAFLD and DM comorbidity, we used the MCODE algorithm in Metascape to construct a modular network of 58 intersected genes to identify core treatment targets (Figure 3A). Through topological network analysis, we found that the key pathways involved include cancer pathways, endocrine resistance, diabetic cardiomyopathy, etc. (Figure 3B). In order to elucidate the relationship between the target and the path, 2 subsets were generated, in which the key genes involved in the first subset were identified. It includes EGFR, AKT1, ESR1, and HSP90AA1, and the main pathways involved are cancer pathways and endocrine resistance, as shown in Figure 3C and Table 3.

**TABLE 3:** Pathway enrichment results for MCODE1 and MCODE2.

Color	MCODE	GO	Description	Log_10_(*p*)
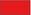	MCODE1	hsa01522	Endocrine resistance	−11.2
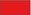	MCODE1	hsa05200	Pathways in cancer	−9.7
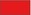	MCODE1	hsa05207	Chemical carcinogenesis‐receptor activation	−9.5
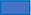	MCODE2	hsa04910	Insulin signaling pathway	−8.2
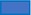	MCODE2	hsa04151	Pl3K‐Akt signaling pathway	−6.5
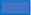	MCODE2	hsa01521	EGFR tyrosine kinase inhibitor resistance	−6.5

### 3.6 The Network of “Compounds‐Targets‐Pathway”

**FIGURE 4:**
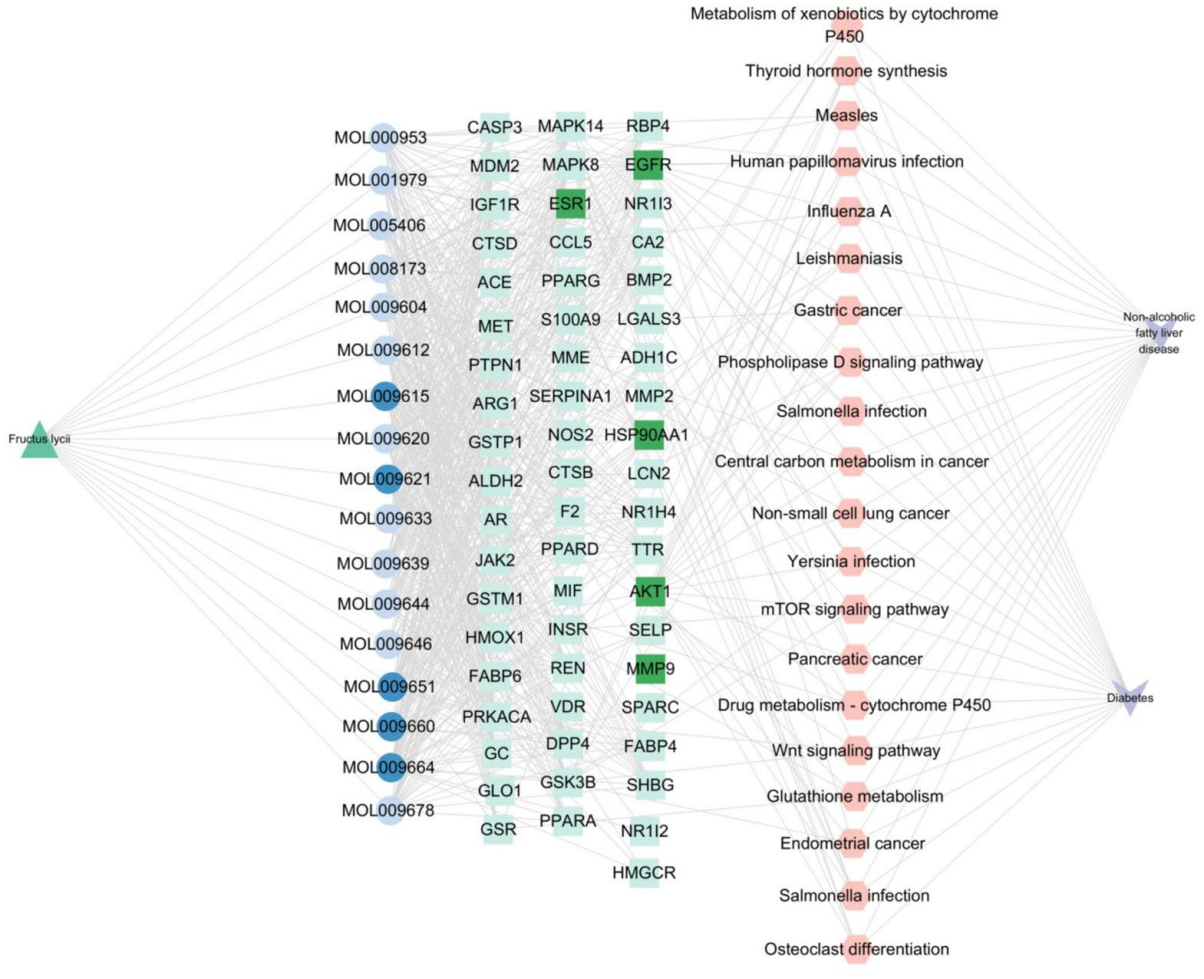
The network of “The active components of LF‐targets‐pathway”.

This network integrated the active components of LF, the overlapping targets of NAFLD and DM comorbidity, and the top 20 pathways from KEGG enrichment analysis. The network was built using Cytoscape 3.9.1 and consists of 98 nodes and 603 edges. This network model revealed how the active components of LF act on specific targets (e.g., proteins, enzymes, receptors) to modulate specific biological pathways (e.g., signaling pathways, metabolic pathways), thereby exerting pharmacological effects. In Figure 4, the blue circle represents the active ingredient of LF, the pink regular hexagon represents the pathway, the green square represents the intersection target gene of LF and NAFLD and DM diseases, the green triangle represents LF, and the purple V‐shape represents NAFLD and DM. The network is topologically analyzed with the built‐in CytoNCA plugin. We found that LF active ingredient cryptoxanthin monoepoxide, 24‐methylenelanost‐8‐enol, 24‐ethylenecycloartan‐3beta,21‐diol, Physalin A, methyl(1R,4aS,7R,7aS)‐4a,7‐dihydroxy‐7‐methyl‐1‐[(2S,3R,4S,5S,6R)‐3,4,5‐trihydroxy‐6‐(hydroxymethyl)oxan‐2‐yl]oxy‐1,5,6,7a‐tetrahydrocyclopenta[d]pyran‐4‐carboxylate ranks relatively high among many active ingredients and is the main core ingredient (Table 4). It can be preliminarily believed that the pharmacological effect of LF is mainly through this active ingredient. EGFR, AKT1, HSP90AA1, ESR1, MMP9 potential pathways of interaction with most genes, including the MAPK, P13K–Akt, FoxO, mTOR, and Phospholipase D signaling pathways, suggesting that these genes may have a role in the treatment of NAFLD and DM comorbidity.

**TABLE 4:** Core component screening of LF.

No.	Compounds	Degree	Betweenness	Closeness
1	MOL009621	175	30486.926	0.57383180
2	MOL009664	118	10852.937	0.47303542
3	MOL009651	112	5146.823	0.46444780
4	MOL009660	109	23675.271	0.45481482
5	MOL009615	108	3826.3154	0.45889387
6	MOL009612	97	2646.528	0.44428363
7	MOL009678	92	1917.0853	0.43669987
8	MOL005406	91	11413.421	0.43300423
9	MOL009620	90	1762.5238	0.43546098
10	MOL001979	90	1854.1022	0.43546098
11	MOL009639	88	1570.98	0.43300423
12	MOL009633	88	1570.98	0.43300423
13	MOL008173	88	1685.7924	0.43300423
14	MOL009644	86	1653.3066	0.42937064
15	MOL000953	84	1582.3344	0.42817295
16	MOL009646	82	8606.51	0.42344826
17	MOL009604	65	939.37744	0.40554821

### 3.7 Bioactives and Drug‐Likeness Property of the Core Components of LF

By conducting ADME analysis on the five core components and applying Lipinski's rules, it was determined that 24‐methylenelanost‐8‐enol, 24‐ethylenecycloartan‐3beta, 21‐diol, and cryptoxanthin monoepoxide are suitable for evaluation as potential new drugs based on their pharmacokinetic parameters. However, Physalin A and methyl(1R,4aS,7R,7aS)‐4a,7‐dihydroxy‐7‐methyl‐1‐[(2S,3R,4S,5S,6R)‐3,4,5‐trihydroxy‐6‐(hydroxymethyl)oxan‐2‐yl]oxy‐1,5,6 7a‐tetrahydrocyclopenta[d]pyran‐4‐carboxylate did not meet the required molecular weight and topological polar surface area criteria for membrane transmembrane, rendering them unsuitable for further evaluation (Table 5).

**TABLE 5:** Bioactives and drug‐likeness property of the core components.

No.	Compounds	Lipinski rules				Lipinski's violations	Bioavailability score	TPSA (Å^2^)
		MW	HBA	HBD	MLog *p*			
		< 500	< 10	≤ 5	< 4.15	≤ 1	> 0.1	< 140
1	MOL009621	440.74	1	1	7.01	1	0.55	20.23
2	MOL009651	568.87	2	1	7.03	0	0.17	32.76
3	MOL009615	456.74	2	2	6.19	0	0.17	40.46
4	MOL009664	526.53	10	3	0.59	1	0.55	156.66
5	MOL009660	406.38	11	6	−2.52	0	0.11	175.37

### 3.8 Molecular Docking

**FIGURE 5:**
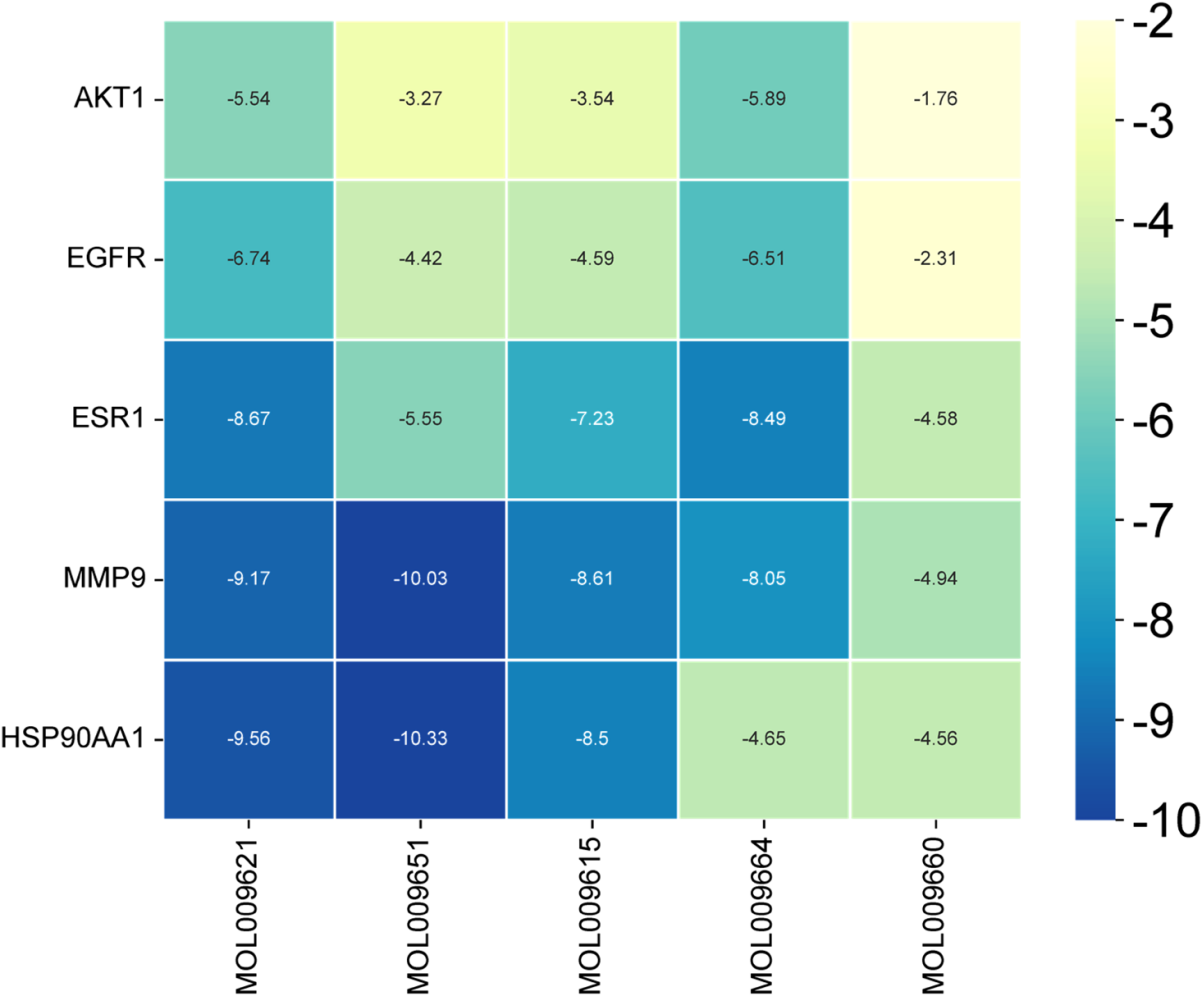
Docking results of core active ingredients and key node proteins.

**FIGURE 6:**
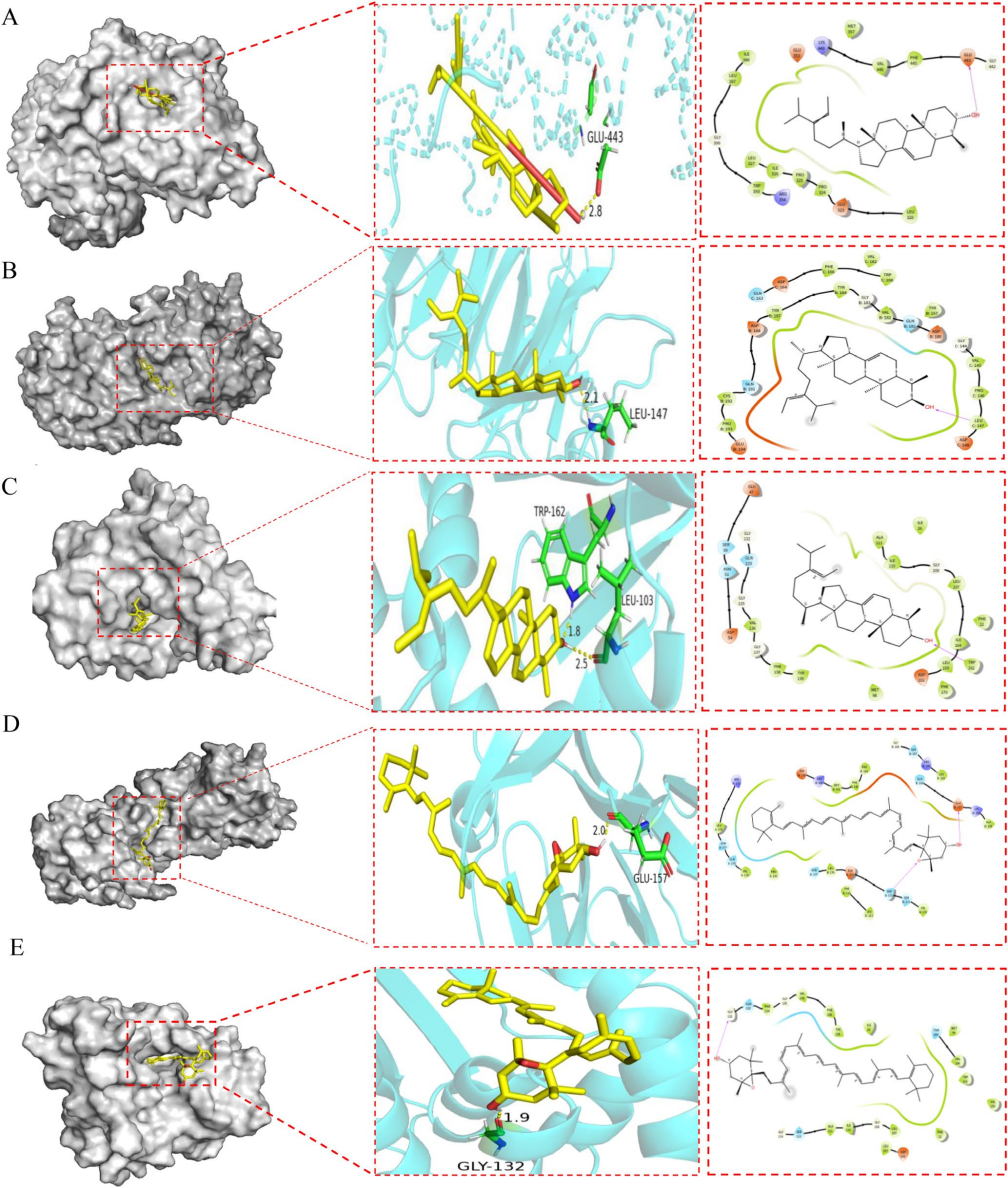
Molecular docking results (A) ESR1 with 24‐methylenelanost‐8‐enol, (B) MMP9 with 24‐methylenelanost‐8‐enol, (C) HSP90AA1 with 24‐methylenelanost‐8‐enol, (D) MMP9 with cryptoxanthin monoepoxide, and (E) HSP90AA1 with cryptoxanthin monoepoxide.

Molecular docking was performed to validate the interactions between the compounds 24‐methylenelanost‐8‐enol and cryptoxanthin monoepoxide with six core proteins (AKT1, EGFR, ESR1, MMP9, HSP90AA1, CASP3). Binding energies between the small molecules and proteins were calculated, as lower binding energies indicate stronger interactions and higher affinity between the ligand and receptor. Most of the binding energies were found to be below −4 kcal/mol, as summarized in Figure 5. In general, binding affinity values greater than −5 kcal/mol indicate no significant binding, values between −5 and −7 kcal/mol suggest moderate binding, and values below −7 kcal/mol reflect strong binding affinity (Pantsar and Poso 2018). The results demonstrated that the binding energies of cryptoxanthin monoepoxide and 24‐methylenelanost‐8‐enol with ESR1, MMP9, and HSP90AA1 were particularly low (< −8 kcal/mol), indicating a strong affinity between these compounds and the target proteins. Interface diagrams of these interactions were created for surface and interior visualization (Figure 6). In this study, the ligands were located within the binding pockets of the target proteins and formed significant interactions with several key amino acid residues. Specifically, during the molecular docking of ESR1 with 24‐methylenelanost‐8‐enol, Glu443 served as a key amino acid residue, interacting strongly with the ligand. The carboxyl group of Glu443 acts as a hydrogen bond acceptor, facilitating the formation of a hydrogen bond with the ligand. The hydrogen bond distance is 2.8 Å, indicating a relatively strong interaction between Glu443 and the ligand (Figure 6A). In the molecular docking of MMP9 with 24‐methylenelanost‐8‐enol, Leu147 forms a notable hydrophobic interaction with the ligand. As a hydrophobic amino acid, the side chain of Leu147 interacts with the hydrophobic portion of the ligand via van der Waals forces. The interaction distance between Leu147 and the ligand is 2.1 Å, suggesting a strong and direct interaction (Figure 6B). In the molecular docking of HSP90AA1 with 24‐methylenelanost‐8‐enol, Trp162, an aromatic amino acid residue, interacts with the ligand's aromatic ring through *π*–*π* stacking interactions. Additionally, Leu103 engages in hydrophobic interactions with the ligand, where the hydrophobic side chain of Leu103 interacts with the ligand's hydrophobic regions through van der Waals forces. The interaction distances between Trp162 and the ligand (1.8 Å) and between Leu103 and the ligand (2.5 Å) further demonstrate strong, direct interactions (Figure 6C). In the molecular docking analysis of MMP9 with cryptoxanthin monoepoxide, Glu157 forms a clear hydrogen bond interaction with the ligand, with the carboxyl group of Glu157 acting as a hydrogen bond acceptor. The interaction distance between Glu157 and the ligand is 2.0 Å, indicating a strong and direct interaction (Figure 6D). In the molecular docking of HSP90AA1 with cryptoxanthin monoepoxide, Gly132 participates in hydrogen bonding with the ligand, with the hydrogen atoms of glycine (Gly) contributing to hydrogen bond formation. The interaction distance between Gly132 and the ligand is 1.9 Å, suggesting a strong and direct interaction (Figure 6E).

### 3.9 Molecular Dynamics Simulations

Molecular dynamics simulations were conducted to evaluate the stability of the receptor‐ligand complexes under physiological conditions. Based on their binding affinities, interactions, and structural diversity, the 24‐methylenelanost‐8‐enol and cryptoxanthin monoepoxide compounds were selected for simulations with ESR1, MMP9, and HSP90AA1. The root mean square deviation (RMSD) was tracked over 100 ns to assess the stability of the complexes. Additionally, root mean square fluctuation (RMSF) analysis was used to measure protein flexibility, providing insights into the dynamic stability of amino acid residues. Typically, a stable complex shows RMSD fluctuations of less than 0.2 nm.

**FIGURE 7:**
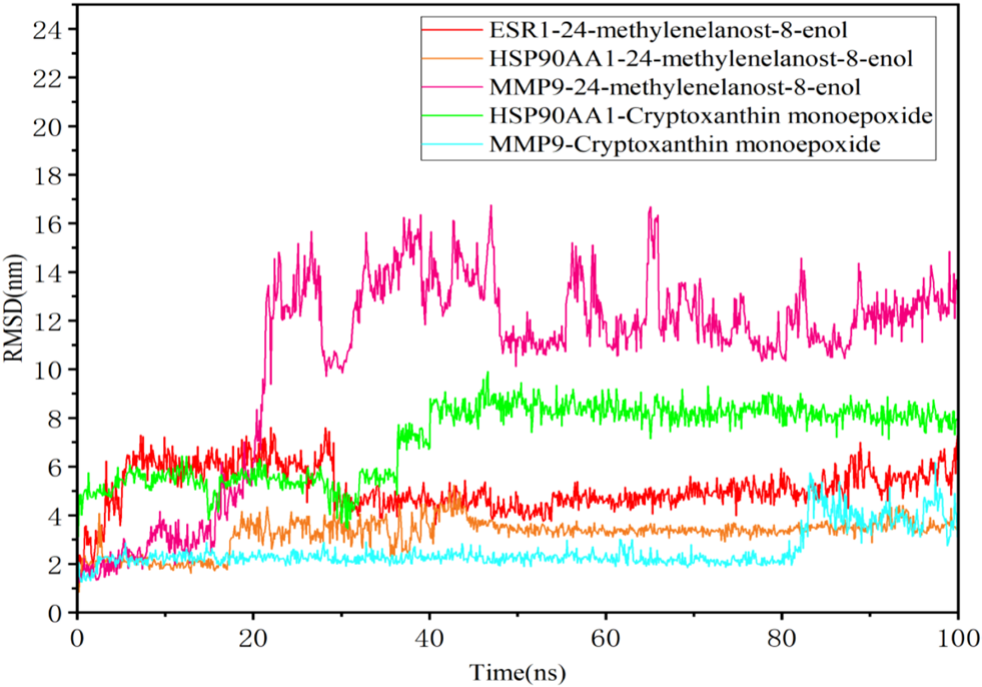
The fluctuation plot of the target protein‐ligand complexes RMSD.

**FIGURE 8:**
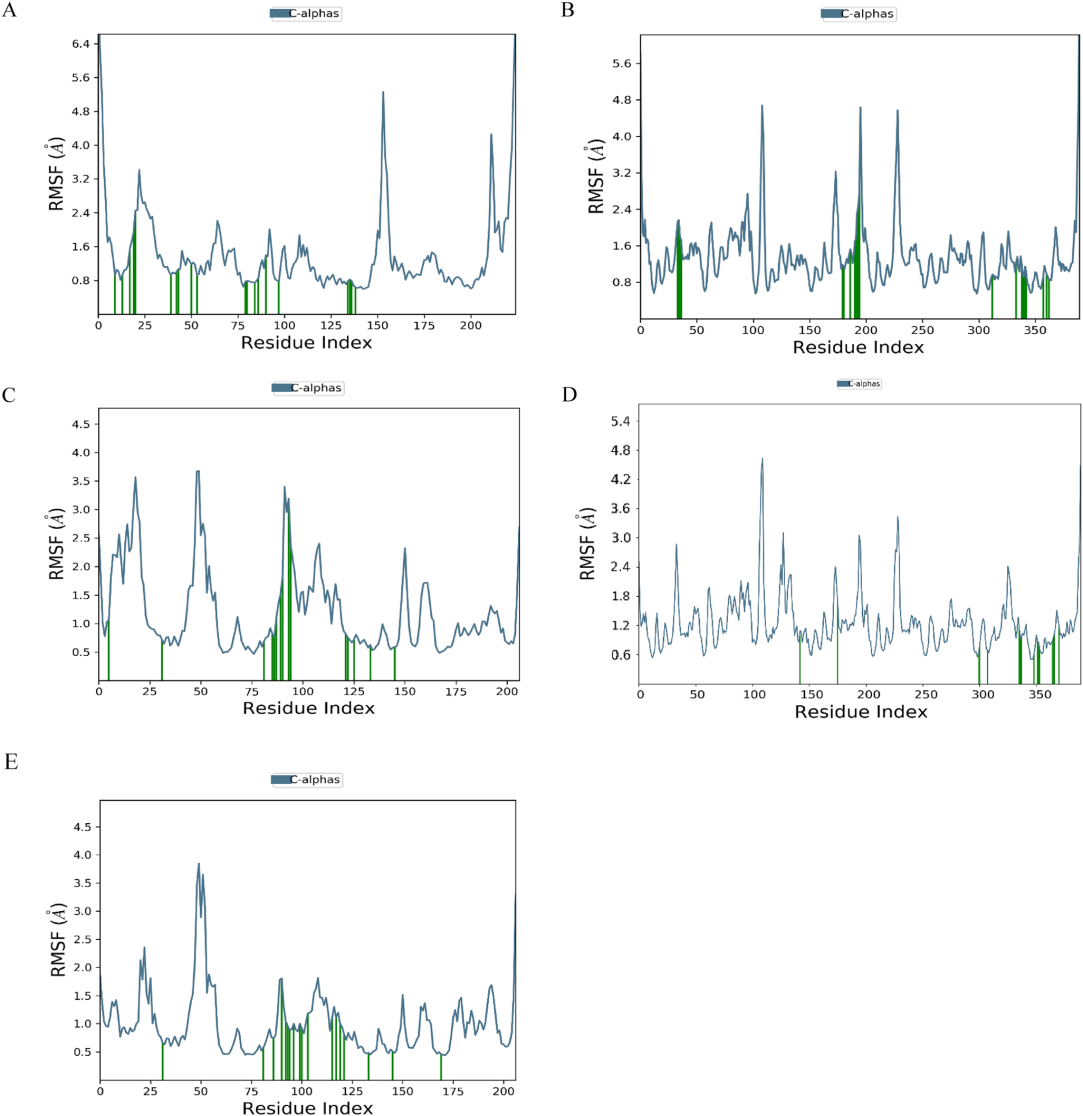
The fluctuation plot of the target protein‐ligand complexes RMSF. (A) ESR1 with 24‐methylenelanost‐8‐enol, (B) MMP9 with 24‐methylenelanost‐8‐enol, (C) HSP90AA1 with 24‐methylenelanost‐8‐enol, (D) MMP9 with cryptoxanthin monoepoxide, and (E) HSP90AA1 with cryptoxanthin monoepoxide. Residues in contact with the ligand are marked in green.

As shown in Figure 7, the RMSD curves of the five protein‐ligand complexes remained stable throughout the simulation, indicating low fluctuation and consistent stability. Specifically, the HSP90AA1 with 24‐methylenelanost‐8‐enol and MMP9 with cryptoxanthin monoepoxide complexes showed stable trajectories across the 0–100 ns time frame. RMSF analysis indicated higher fluctuations at the beginning and end of the simulation, suggesting some initial and final instability in the proteins. Residues in direct contact with the ligands, highlighted in green, exhibited lower RMSF values, indicating enhanced stability due to strong interactions with the ligands. Notably, the HSP90AA1 with cryptoxanthin monoepoxide complex displayed reduced residue fluctuation, indicating increased rigidity in these regions and suggesting that cryptoxanthin monoepoxide effectively stabilizes the target proteins (Figure 8).

**FIGURE 9:**
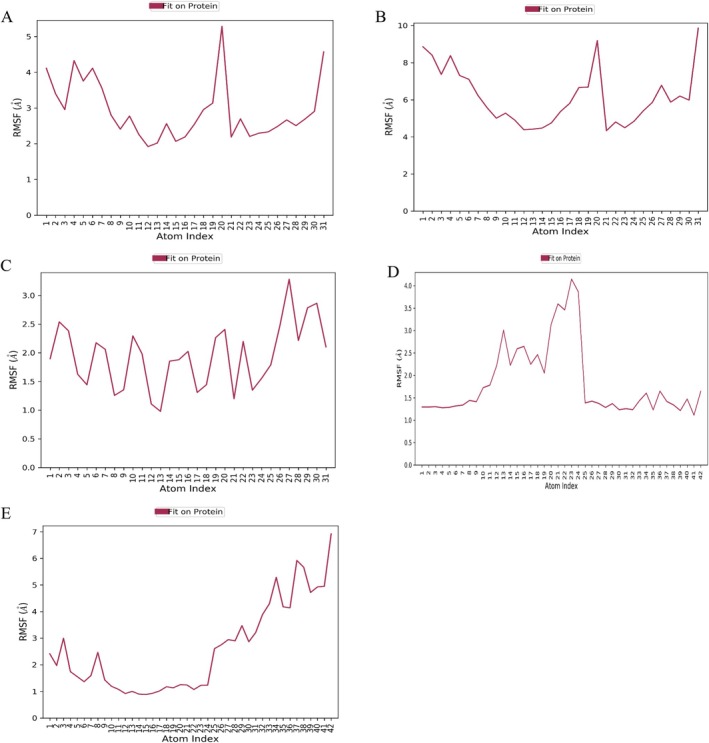
Hydrogen bond number of complex. (A) ESR1with 24‐methylenelanost‐8‐enol, (B) MMP9 with 24‐methylenelanost‐8‐enol, (C) HSP90AA1 with 24‐methylenelanost‐8‐enol, (D) MMP9 with cryptoxanthin monoepoxide, and (E) HSP90AA1 with cryptoxanthin monoepoxide.

The interactions between 24‐methylenelanost‐8‐enol, cryptoxanthin monoepoxide, and the target proteins involved various binding mechanisms, including hydrogen bonds, water bridges, and hydrophobic interactions. Hydrogen bonding, a crucial non‐covalent interaction, was particularly notable. During the 100 ns simulation, the ESR1 with 24‐methylenelanost‐8‐enol complex formed 0–1 hydrogen bonds, the MMP9 with 24‐methylenelanost‐8‐enol complex formed 0–2 bonds, the HSP90AA1 with 24‐methylenelanost‐8‐enol complex formed 0–3 bonds, and the MMP9 with cryptoxanthin monoepoxide and HSP90AA1 with cryptoxanthin monoepoxide complexes formed 0–3 and 0–2 bonds, respectively (Figure 9). Residues involved in these interactions, such as GLU‐157, SER‐171, and SER‐172, are shown in Figure S1.

**FIGURE 10:**
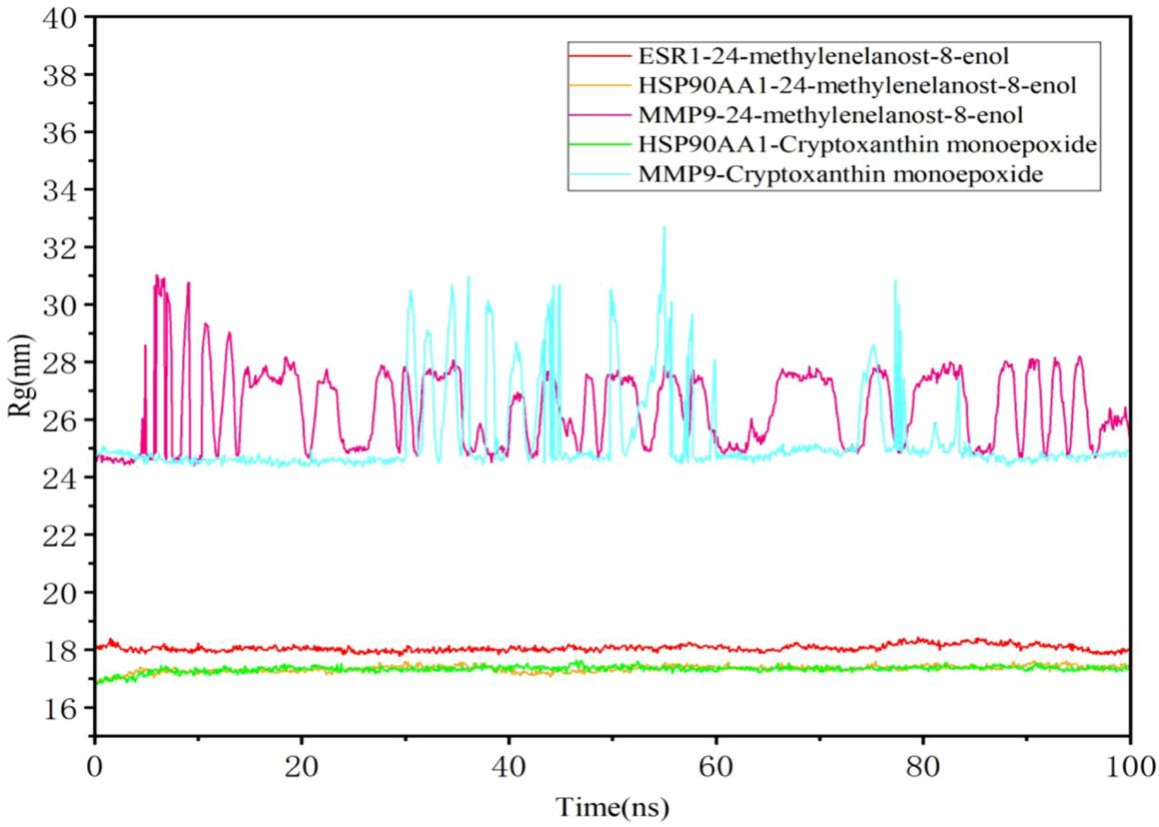
Compactness of the protein according to Rg.

The RMSF of the ligands also remained low when binding was stable. Figure S2 demonstrates the stability of the HSP90AA1 with 24‐methylenelanost‐8‐enol, MMP9 with cryptoxanthin monoepoxide, and HSP90AA1 with cryptoxanthin monoepoxide complexes. The radius of gyration (Rg), reflecting the compactness and flexibility of the ligand‐protein complex, was used to further assess stability. A higher Rg value correlates with increased ligand flexibility and decreased stability. As shown in Figure 10, the Rg of the HSP90AA1 with cryptoxanthin monoepoxide and HSP90AA1 with 24‐methylenelanost‐8‐enol complexes remained stable, further confirming the binding stability of these complexes.

### 3.10 Results of Mendelian Randomization Analysis

We performed MR analysis to explore the causal relationship between six key genes (EGFR, AKT1, ESR1, MMP9, HSP90AA1, CASP3) and two diseases: NAFLD and DM.

**FIGURE 11:**
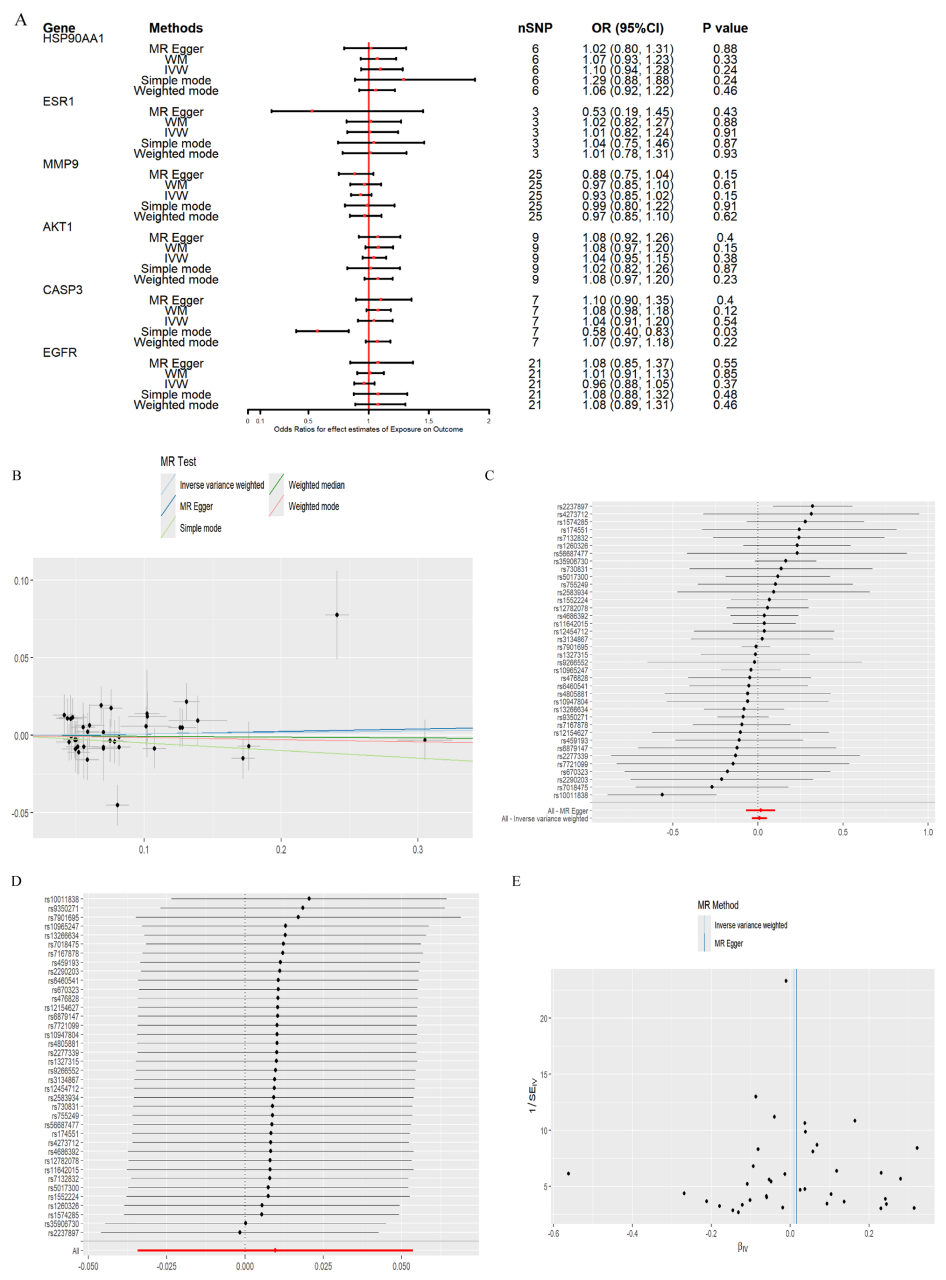
Results of MR analysis of key genes on DM (A) MR analysis results of different genes and DM, (B) Causal effect scatterplot of MR analysis of the relationship between CASP3 and DM, (C) Causal effect forest plot of each SNP in CASP3 on DM, (D) Sensitivity analysis of CASP3 and DM, and (E) CASP3 and DMr relationship SNP biased funnel plot.

In the MR analysis for DM, CASP3 showed correlation with DM. In the Simple Mode analysis, CASP3 had an OR = 0.58 (95% CI: 0.40–0.83), *p* value = 0.03, suggesting that higher levels of CASP3 may be associated with DM. This indicates that CASP3 may play a role in DM pathogenesis. However, other MR methods, such as IVW and MR‐Egger, did not show a significant causal relationship for CASP3. Despite this, the Simple Mode results still indicate a potential correlation. Sensitivity analyses further confirmed the robustness of these findings, as there was no significant heterogeneity or pleiotropic interference. Cochran's *Q* test showed no significant heterogeneity in CASP3's effect on DM. Additionally, the MR‐Egger regression intercept was 0.0114 (*p* = 0.4147), suggesting no significant horizontal pleiotropy. For the other genes, no significant causal relationships were found with DM, as neither IVW, MR‐Egger, nor weighted median analysis reached statistical significance, implying a weak association (Figure 11, Table 6).

**TABLE 6:** Heterogeneity and pleiotropy of MR analysis results of DM.

Gene	Heterogeneity				Pleiotropy		MR‐PRESSO	
	MR‐Egger		IVW		MR‐Egger		Global test	
	Cochran's *Q*	*p*	Cochran's *Q*	*p*	Egger intercept	*p*	RSSobs	*p*
AKT1	10.55	0.0145	13.68	0.0084	0.0114	0.4147	NA	NA
EGFR	33.69	0.0137	34.40	0.0165	0.0032	0.5469	21.86489	0.141
ESR1	NA	NA	NA	NA	NA	NA	NA	NA
MMP9	2.15	0.1430	2.43	0.2969	0.0122	0.7780	36.9213	0.046
HSP90AA1	17.27	0.1396	18.55	0.1376	0.0143	0.3639	18.38011	0.178
CASP3	10.55	0.0145	13.68	0.0084	0.0114	0.4147	NA	NA

**FIGURE 12:**
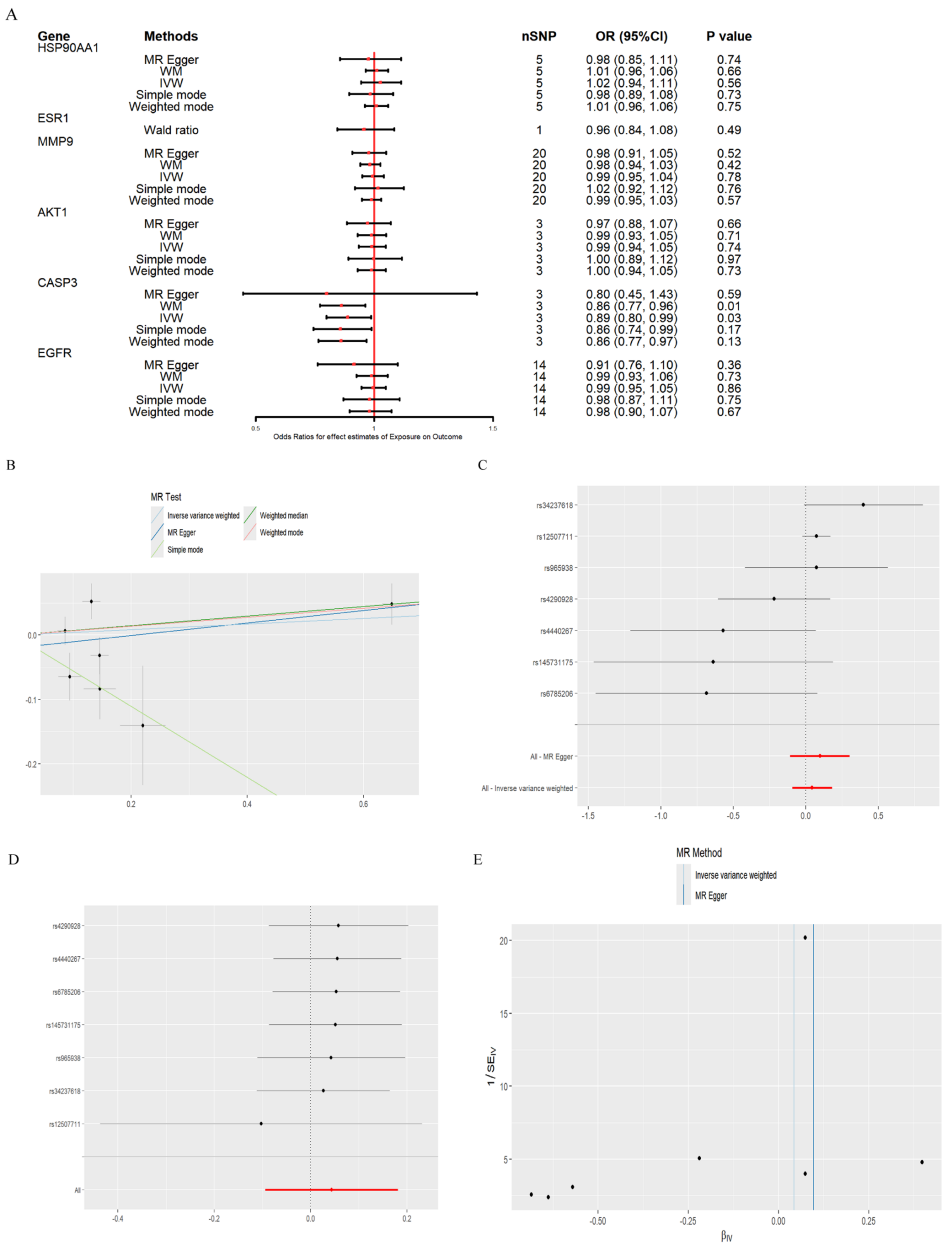
Results of MR analysis of key genes for NAFLD (A) MR analysis results of different genes and NAFLD, (B) Scatterplot of causal effect of MR analysis of the relationship between CASP3 and NAFLD, (C) Forest plot of causal effect of each SNP in CASP3 on NAFLD, (D) Sensitivity analysis of CASP3 and NAFLD, and (E) CASP3 and NAFLD relationship SNP‐biased funnel plot.

In the IVW analysis for NAFLD, CASP3 showed a significant association, with OR = 0.89 (95% CI: 0.80–0.99), *p* = 0.03. This suggests that higher levels of CASP3 may be associated with NAFLD, indicating its potential role in the pathogenesis of NAFLD. To validate this causal relationship, we conducted sensitivity analyses, which revealed no significant heterogeneity or pleiotropic interference. Cochran's *Q* test showed no significant heterogeneity (*p* = 0.02), and the MR‐Egger intercept was −0.0201 (*p* = 0.51), indicating no significant pleiotropic effect on the causal relationship. These analyses suggest a significant and robust causal relationship between CASP3 and NAFLD. The other genes did not show significant causal relationships with NAFLD, as neither IVW, MR‐Egger, nor weighted median analysis reached statistical significance, indicating a weak or nonexistent association (Figure 12).

Our MR analysis suggested that CASP3 may have a causal relationship with both DM and NAFLD, particularly in NAFLD, where the effect appears relatively significant. However, no significant causal relationships were observed for other genes, including HSP90AA1, MMP9, AKT1, and EGFR, in relation to DM and NAFLD. All analyses underwent rigorous sensitivity tests, heterogeneity, and pleiotropy tests, which confirmed that the causality of CASP3 remains statistically robust (Table 7).

**TABLE 7:** Heterogeneity and Pleiotropy of MR Analysis results of NAFLD.

Gene	Heterogeneity				Pleiotropy		MR‐PRESSO	
	MR‐Egger		IVW		MR‐Egger		Global test	
	Cochran's *Q*	*p*	Cochran's *Q*	*p*	Egger intercept	*p*	RSSobs	*p*
AKT1	6.39	0.17	7.27	0.20	0.0156	0.50	5.44	0.877
EGFR	25.50	0.14	26.87	0.14	−0.0211	0.32	29.58	0.143
ESR1	0.22	0.64	1.88	0.39	0.1039	0.42	NA	NA
MMP9	13.94	0.93	14.59	0.93	0.0073	0.43	18.76	0.87
HSP90AA1	6.39	0.17	7.27	0.20	0.0156	0.50	17.94	0.38
CASP3	13.32	0.02	14.67	0.02	−0.0201	0.51	27.87	0.27

## 4 Discussion

Globally, NAFLD and its advanced form, non‐alcoholic steatohepatitis (NASH), affect approximately 56% and 37% of type 2 diabetes patients, respectively. The coexistence of diabetes and NAFLD substantially elevates the risk of advanced liver fibrosis (Younossi et al. 2016). As fibrosis progresses, all‐cause mortality among NAFLD patients increases (Dulai et al. 2017). The pathophysiology linking NAFLD and diabetes is complex, involving multiple mechanisms, which makes single‐component treatments less effective and more prone to adverse effects. Current drugs offer limited efficacy in treating both NAFLD and diabetes and often raise safety concerns.

LF contains various active compounds, including polysaccharides, polyphenols, and carotenoids, known for their diverse pharmacological effects, such as antioxidant, anti‐inflammatory, antitumor, neuroprotective, and immune‐enhancing properties (Toh et al. 2021; Chen et al. 2018). Recently, the potential of LF in treating NAFLD and DM has gained significant attention. Zhou et al. demonstrated that LBPs have an inhibitory effect on acute experimental diabetes in mice (Zhou et al. 2023). Transplantation of LBP‐mediated gut microbiota showed a protective effect on fasting blood glucose in diabetic mice (Zhou et al. 2022). Additionally, glucan‐type LBPs have been found to reduce liver inflammation and damage by downregulating the harmful bacteria enterococcus and its metabolite lipopolysaccharide (LPS) in high‐fat diet‐induced NAFLD rats, thereby inhibiting the activation of the LPS/TLR4/NF‐κB signaling pathway (Gao et al. 2021). Despite these promising findings, the precise pharmacological mechanisms of LF in treating the comorbidity of NAFLD and DM remain unclear.

Network pharmacology was used to identify 17 active compounds in LF and 1,853 NAFLD and 4,333 DM‐related targets, and finally found a total of 58 targets for LF treatment of NAFLD and DM comorbidity, suggesting that LF affects these conditions through multiple targets. By constructing the composition‐target‐pathway network diagram, we identified the main components of LF for treating NAFLD and DM, including cryptoxanthin monoepoxide, 24‐methylenelanost‐8‐enol, 24‐ethylenecycloartan‐3beta, 21diol, physalin A, and methyl(1R,4aS,7R,7aS)‐4a,7‐dihydroxy‐7‐methyl‐1‐[(2S,3R,4S,5S,6R)‐3,4,5‐trihydroxy‐6‐(hydroxymethyl)oxan‐2‐yl]oxy‐1,5,6,7a‐tetrahydrocyclopenta[d]pyran‐4‐carboxylate. Among these, 24‐ethylenecycloartan‐3beta, 21‐diol, and 24‐methylenelanost‐8‐enol belong to the terpenoid class.

Triterpenes are one of the three major secondary metabolites, which have pharmacological effects, such as antitumor, regulation of blood lipids, protection of liver and immune regulation (Chen 2020; Min et al. 2024; Nazaruk and Borzym‐Kluczyk 2015). Activation of the LKB1/AMPK pathway by terpenoids inhibits fatty acid synthesis, enhances mitochondrial β‐oxidation, and reduces lipase expression, improving non‐alcoholic steatohepatitis (NASH) and preventing NAFLD progression (Omidkhoda et al. 2023). Triterpenoids promote insulin secretion, enhance insulin sensitivity, and inhibit key glucose homeostasis enzymes such as PTP1B and alpha‐glucosidase (Liu et al. 2023). Cryptoxanthin monoepoxide, a common carotenoid and primary source of vitamin A in the body, exhibits antioxidant, immunomodulatory, anticancer, and antiaging properties (Jiao et al. 2019). Cryptoxanthin may enhance insulin sensitivity and improve blood glucose control in diabetic patients by modulating insulin signaling pathways. Additionally, its antioxidant properties protect pancreatic beta cells from oxidative damage, maintaining insulin secretion (Nishino et al. 2021). β‐cryptoxanthin has been shown to improve NAFLD through various pathways, including enhancing insulin resistance, inhibiting oxidative stress and inflammation, and modulating macrophage populations (Nishino et al. 2021).

We found that the key node proteins in the network are primarily HSP90AA1, EGFR, AKT1, MMP9, ESR1, and CASP3. In recent years, HSP90 inhibitors have demonstrated a protective effect on diabetes (Lei et al. 2004; Lazaro et al. 2015). Elevated serum HSP90α levels in patients with type 2 diabetes are associated with oxidative stress and are an independent risk factor for the progression of diabetic vascular disease, providing certain predictive value (Xinyi et al. 2022). Research indicates that EGFR plays a significant role in NAFLD and is a potential therapeutic target. EGFR activation occurs in the liver tissue of high‐fat diet‐induced NAFLD mice (Choung et al. 2019). GO enrichment analysis indicated that LF is primarily involved in the negative regulation of blood pressure and the positive regulation of insulin secretion in response to glucose stimulation. KEGG pathway enrichment analysis revealed that LF influences the MAPK, PI3K‐Akt, and FoxO signaling pathway, contributing to its therapeutic effects on NAFLD and DM comorbidity. Emerging evidence suggests that the ERK1/2 signaling module plays a beneficial role in regulating glucose‐stimulated insulin secretion and promoting β‐cell survival (Kalwat and Thurmond 2013). Yuan et al. (Yuan et al. 2012) found that long‐term insulin stimulation of HepG2 cells reduces insulin signal transduction via the PI3K signaling pathway, leading to insulin resistance. FoxOs have been reported to prevent liver fibrosis by inhibiting the proliferation and transdifferentiation of hepatic stellate cells. Mice lacking liver FoxOs are more susceptible to non‐alcoholic steatohepatitis than wild‐type mice, highlighting the key role of FoxOs in maintaining liver metabolism and cellular homeostasis; dysregulation of FoxOs may be implicated in the development of NAFLD (Dong 2017). The crosstalk between mTOR and its upstream regulatory factors Notch, Hedgehog, and Hippo impacts the occurrence and development of NAFLD‐related hepatocellular carcinoma (Feng et al. 2022). Additionally, mTOR exhibits both antidiabetic and prodiabetic effects (Tuo and Xiang 2019).

Meanwhile, semiflexible molecular docking was performed on the target protein. The docking conformation of computer simulation shows that 24‐methylenelanost‐8‐enol, cryptoxanthin monoepoxide, 24‐ethylenecycloartan‐3beta, and 21‐diol have good binding activity to HSP90AA1, MMP9, and ESR1. Studies have found that triterpenoids (24‐methylenelanost‐8‐enol) and carotenoids (cryptoxanthin monoepoxide) can improve NAFLD and DM comorbidity. We speculate that LF plays a therapeutic role in NAFLD and DM comorbidity through these active compounds, target genes, and signaling pathways. Further exploration of these interactions could pave the way for targeted therapeutic strategies in NAFLD and DM comorbidity. Our MR analysis suggests that CASP3 may have a causal relationship with both T2D and NAFLD consistent with our network pharmacological predictions. As a central mediator of apoptosis, CASP3 plays a pivotal role in regulating cell death (Porter and Jänicke 1999). Additionally, the mTOR, MAPK, and PI3K‐Akt signaling pathways were implicated in the therapeutic mechanisms of LF. These pathways are well‐established for their roles in regulating cell metabolism, inflammation, and apoptosis (Zhang and Liu 2002; Panwar et al. 2023; Su et al. 2023), all of which are closely linked to NAFLD and DM. The involvement of the MAPK pathways, in particular, in both apoptosis and inflammation, further underscores the relevance of our findings, as these processes are crucial to the progression of NAFLD and DM.

This study uncovers the potential molecular mechanisms of LF in treating the comorbidity of NAFLD and DM, though several limitations should be acknowledged. The accuracy and reliability of our compound and target predictions depend on the quality of the databases used. Additionally, the complexity of metabolic diseases like NAFLD and DM highlights the need for experimental validation to confirm the in vivo efficacy of these compounds. To further advance our understanding, we propose the following future directions: (Younossi et al. 2023) isolating and purifying the bioactive compounds identified (terpenoids and cryptoxanthin) from LF, followed by in vitro and in vivo experiments to confirm their pharmacological effects on NAFLD and DM (Mantovani et al. 2020); further exploring the molecular mechanisms underlying the observed effects, with a particular focus on apoptosis, oxidative stress, and inflammation pathways. These efforts will provide deeper insights into how LF compounds influence disease progression at the molecular level.

## 5 Conclusion

The intricate interplay between NAFLD and DM comorbidity involves shared molecular pathways, with emerging clinical and experimental evidence supporting LF as a potential dual‐target therapy. Leveraging integrative computational approaches, network pharmacology, molecular docking, and MR, this study elucidates LF's therapeutic mechanisms. Key terpenoid and cryptoxanthin constituents, including 24‐methylenelanost‐8‐enol and cryptoxanthin monoepoxide, were identified as bioactive agents interacting with pivotal targets such as HSP90AA1 and MMP9. MR analysis further implicated CASP3, a central apoptosis regulator, as a potential causal link between NAFLD and DM. Importantly, LF demonstrated a capacity to modulate the MAPK signaling pathway, attenuating apoptosis and inflammatory cascades. These insights advance the mechanistic understanding of NAFLD‐DM crosstalk and position LF as a promising nutraceutical candidate for mitigating these intertwined metabolic disorders through multi‐target, pathway‐driven interventions.

## Author Contributions

**Peng Sun:** writing – original draft, writing – review and editing. **Jiahui Song:** funding acquisition, investigation. **Yang Liu:** software. **Xiujing Li:** software. **Yiming Zhang:** software. **Yuxing Zhou:** software. **Wei Gong:** software, writing – original draft, writing – review and editing.

## Ethics Statement

The authors have nothing to report.

## Conflicts of Interest

The authors declare no conflicts of interest.

## Supporting information


**Figure S1.** RMSF of ligands (A) ESR1 with 24‐methylenelanost‐8‐enol, (B) MMP9 with 24‐methylenelanost‐8‐enol, (C) HSP90AA1 with 24‐methylenelanost‐8‐enol, (D) MMP9 with cryptoxanthin monoepoxide, and (E) HSP90AA1 with cryptoxanthin monoepoxide.
**Figure S2.** PL‐contacts (A) ESR1with 24‐methylenelanost‐8‐enol, (B) MMP9 with 24‐methylenelanost‐8‐enol, (C) HSP90AA1 with 24‐methylenelanost‐8‐enol, (D) MMP9 with cryptoxanthin monoepoxide, and (E) HSP90AA1 with cryptoxanthin monoepoxide.

**Table S1.** Target genes related active compounds of LF.
**Table S2.** NAFLD‐related genes screened from DisGeNET, GeneCards, OMIM, TTD, DrugBank, and UniProt databases.
**Table S3.** DM‐related genes screened from DisGeNET, GeneCards, OMIM, TTD, DrugBank, and UniProt databases.
**Table S4.** The putative targets of LF against NAFLD and DM comorbidity.
**Table S5.** Protein–protein interaction (PPI) network of overlapping target proteins of LF, NAFLD, and DM comorbidity.
**Table S6.** The GO (MF, BP, and CC) enrichment analysis.
**Table S7.** The enriched KEGG pathways analysis.
**Table S8.** SNPs information for the 6 feature genes.
**Table S9.** Results of MR analysis of feature genes and DM.
**Table S10.** Results of MR analysis of feature genes and NAFLD.

## Data Availability

The datasets used and/or analyzed during the current study are available from the corresponding author on request.
