# Supplementary material for: Investigating the Mechanisms of Lycii fructus in Treating Nonalcoholic Fatty Liver Disease and Diabetes Comorbidity Through Network Pharmacology and Molecular Dynamics
Source: Food Sci Nutr. 2025 May 26;13(6):e70256. doi: 10.1002/fsn3.70256 (PMC12106045; doi:10.1002/fsn3.70256)
Supplement: Supplementary file 1 — Figure S1. RMSF of ligands (A) ESR1 with 24‐methylenelanost‐8‐enol, (B) MMP9 with 24‐methylenelanost‐8‐enol, (C) HSP90AA1 with 24‐methylenelanost‐8‐enol, (D) MMP9 with cryptoxanthin monoepoxide, and (E) HSP90AA1 with cryptoxanthin monoepoxide. Figure S2. PL‐contacts (A) ESR1with 24‐methylenelanost‐8‐enol, (B) MMP9 with 24‐methylenelanost‐8‐enol, (C) HSP90AA1 with 24‐methylenelanost‐8‐enol, (D) MMP9 with cryptoxanthin monoepoxide, and (E) HSP90AA1 with cryptoxanthin monoepoxide. [file FSN3-13-e70256-s002.docx]

**Supplementary Figures**

Fig.S1 RMSF of ligands (A) ESR1-24-methylenelanost-8-enol (B) MMP9-24-methylenelanost-8-enol (C) HSP90AA1-24-methylenelanost-8-enol (D) MMP9-Cryptoxanthin monoepoxide (E) HSP90AA1-Cryptoxanthin monoepoxide.

Fig.S2 PL-Contacts (A) ESR1-24-methylenelanost-8-enol (B) MMP9-24-methylenelanost-8-enol (C) HSP90AA1-24-methylenelanost-8-enol (D) MMP9-Cryptoxanthin monoepoxide (E) HSP90AA1-Cryptoxanthin monoepoxide.


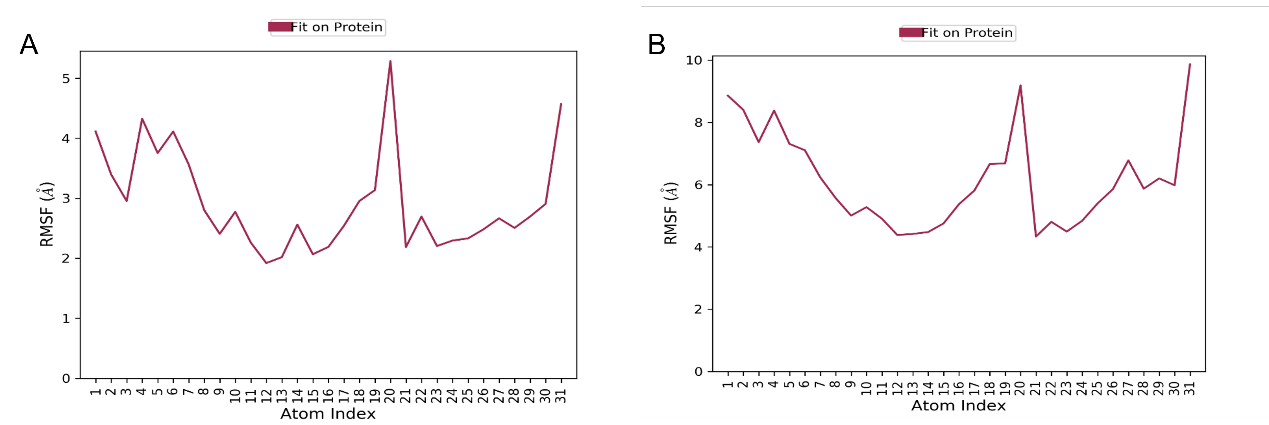


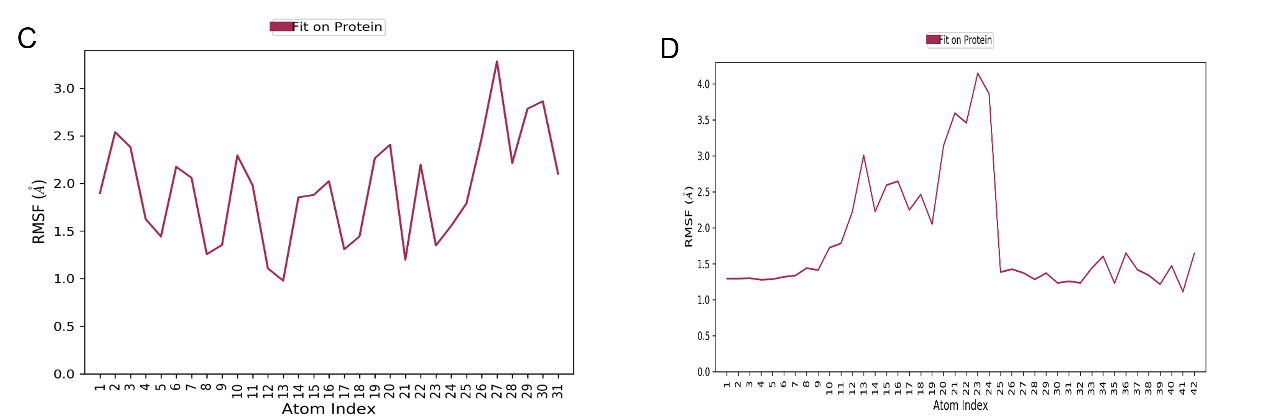


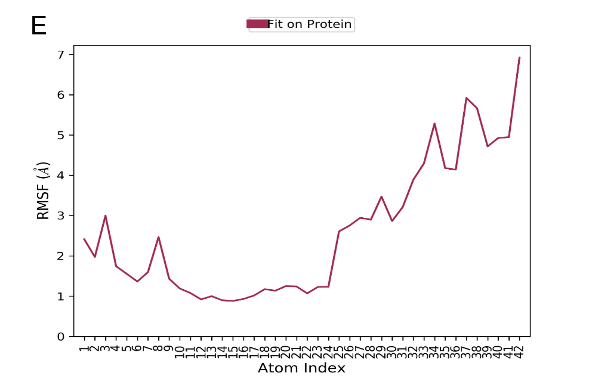


Fig.S1 RMSF of ligands (A) ESR1-24-methylenelanost-8-enol

1. MMP9-24-methylenelanost-8-enol (C) HSP90AA1-24-methylenelanost-8-enol

(D) MMP9-Cryptoxanthin monoepoxide (E) HSP90AA1-Cryptoxanthin monoepoxide.


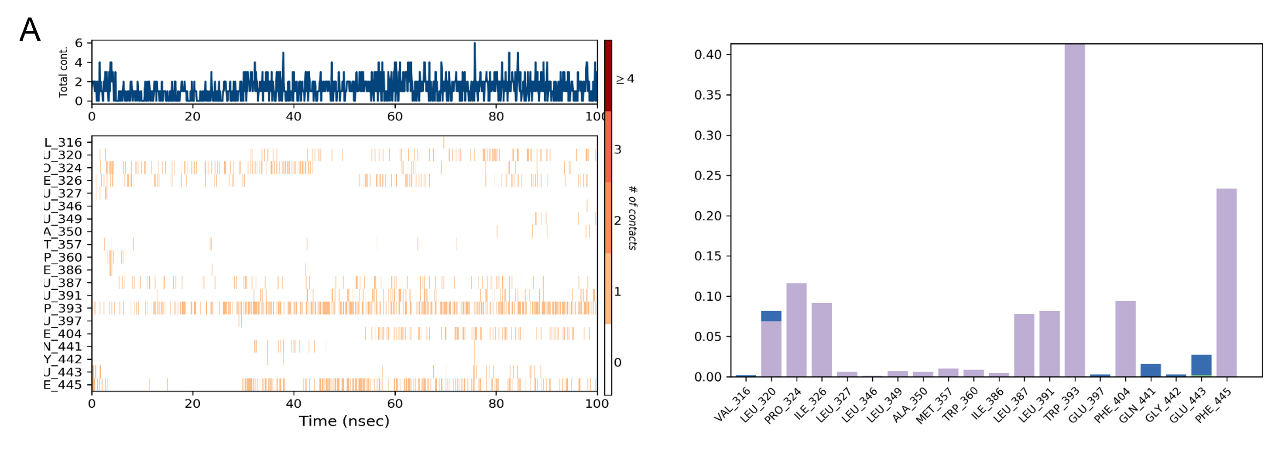


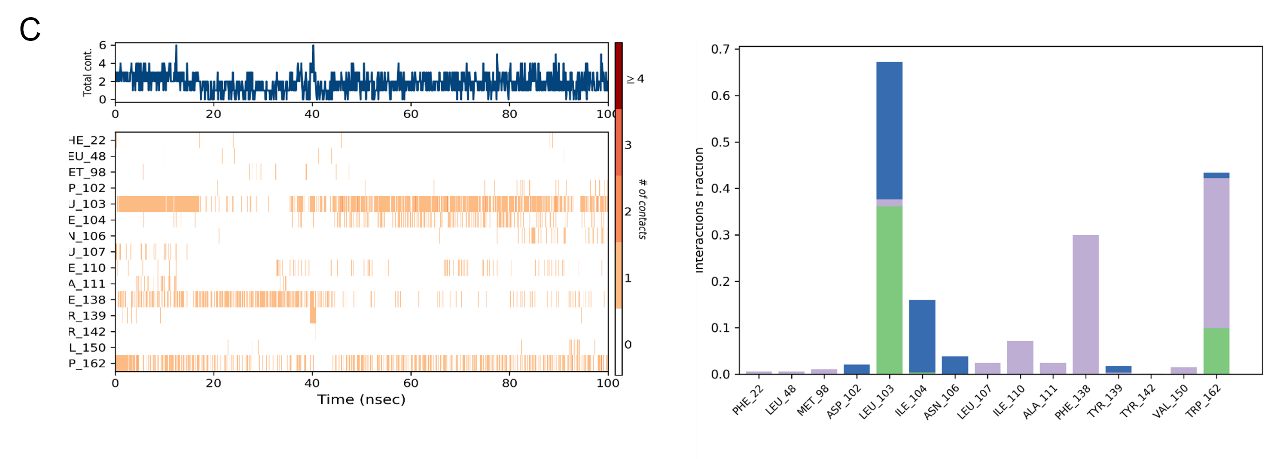

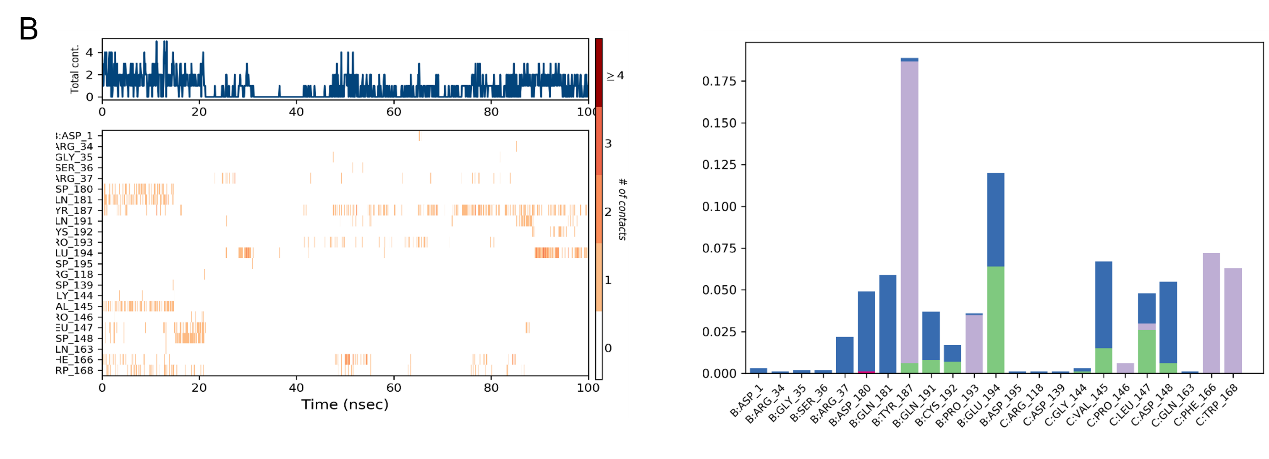


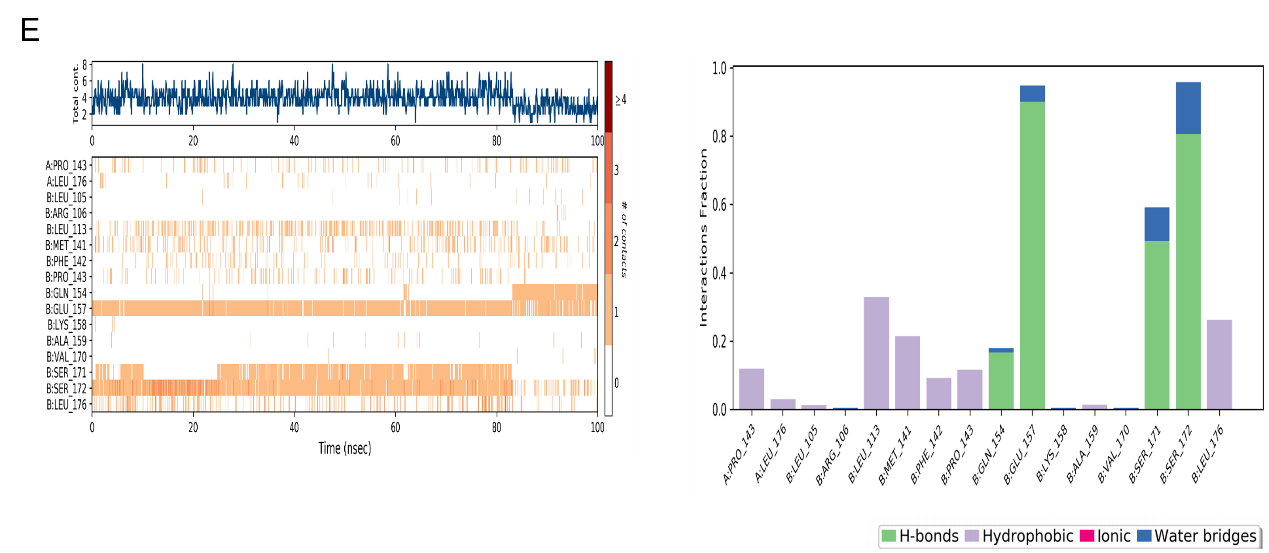

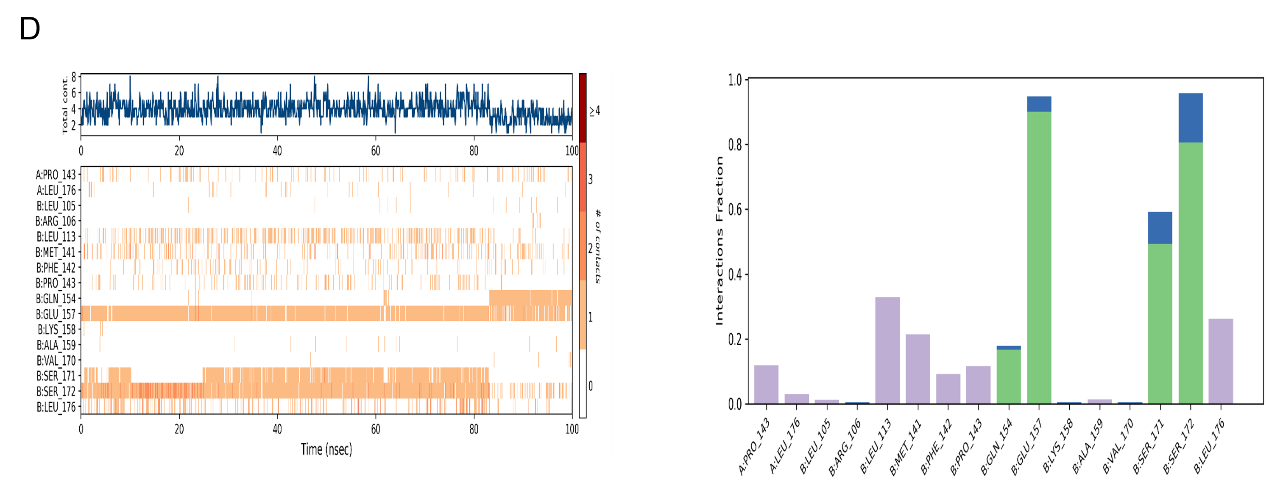


Fig.S2 PL-Contacts (A) ESR1-24-methylenelanost-8-enol

1. MMP9-24-methylenelanost-8-enol (C) HSP90AA1-24-methylenelanost-8-enol

(D) MMP9-Cryptoxanthin monoepoxide (E) HSP90AA1-Cryptoxanthin monoepoxide.
