# Supplementary material for: Investigating the Mechanisms of Lycii fructus in Treating Nonalcoholic Fatty Liver Disease and Diabetes Comorbidity Through Network Pharmacology and Molecular Dynamics
Source: Food Sci Nutr. 2025 May 26;13(6):e70256. doi: 10.1002/fsn3.70256 (PMC12106045; doi:10.1002/fsn3.70256)
Supplement: Supplementary file 2 — Table S1. Target genes related active compounds of LF. Table S2. NAFLD‐related genes screened from DisGeNET, GeneCards, OMIM, TTD, DrugBank, and UniProt databases. Table S3. DM‐related genes screened from DisGeNET, GeneCards, OMIM, TTD, DrugBank, and UniProt databases. Table S4. The putative targets of LF against NAFLD and DM comorbidity. Table S5. Protein–protein interaction (PPI) network of overlapping target proteins of LF, NAFLD, and DM comorbidity. Table S6. The GO (MF, BP, and CC) enrichment analysis. Table S7. The enriched KEGG pathways analysis. Table S8. SNPs information for the 6 feature genes. Table S9. Results of MR analysis of feature genes and DM. Table S10. Results of MR analysis of feature genes and NAFLD. [file FSN3-13-e70256-s001.zip › Supporting Tables/Table S10.docx]

**Table S10 Results of MR Analysis of feature genes and NAFLD**

| Gene | ID number | MR Methods | N SNPs | OR (95%CI) | *P* value |
| --- | --- | --- | --- | --- | --- |
|  |  | MR Egger | 6 | 1.02 (0.80, 1.31) | 0.88 |
|  |  | Weighted median | 6 | 1.07 (0.93, 1.23) | 0.33 |
| HSP90AA1 | eqtl-a-ENSG00000080824 | Inverse variance weighted | 6 | 1.10 (0.94, 1.28) | 0.24 |
|  |  | Simple mode | 6 | 1.29 (0.88, 1.88) | 0.24 |
|  |  | Weighted mode | 6 | 1.06 (0.92, 1.22) | 0.46 |
|  |  | MR Egger | 3 | 0.53 (0.19, 1.45) | 0.43 |
|  |  | Weighted median | 3 | 1.02 (0.82, 1.27) | 0.88 |
| ESR1 | eqtl-a-ENSG00000091831 | Inverse variance weighted | 3 | 1.01 (0.82, 1.24) | 0.91 |
|  |  | Simple mode | 3 | 1.04 (0.75, 1.46) | 0.87 |
|  |  | Weighted mode | 3 | 1.01 (0.78, 1.31) | 0.93 |
|  |  | MR Egger | 25 | 0.88 (0.75, 1.04) | 0.15 |
|  |  | Weighted median | 25 | 0.97 (0.85, 1.10) | 0.61 |
| MMP9 | eqtl-a-ENSG00000100985 | Inverse variance weighted | 25 | 0.93 (0.85, 1.02) | 0.15 |
|  |  | Simple mode | 25 | 0.99 (0.80, 1.22) | 0.91 |
|  |  | Weighted mode | 25 | 0.97 (0.85, 1.10) | 0.62 |
|  |  | MR Egger | 9 | 1.08 (0.92, 1.26) | 0.40 |
|  |  | Weighted median | 9 | 1.08 (0.97, 1.20) | 0.15 |
| AKT1 | eqtl-a-ENSG00000142208 | Inverse variance weighted | 9 | 1.04 (0.95, 1.15) | 0.38 |
|  |  | Simple mode | 9 | 1.02 (0.82, 1.26) | 0.87 |
|  |  | Weighted mode | 9 | 1.08 (0.97, 1.20) | 0.23 |
|  |  | MR Egger | 7 | 1.10 (0.90, 1.35) | 0.40 |
|  |  | Weighted median | 7 | 1.08 (0.98, 1.18) | 0.12 |
| CASP3 | eqtl-a-ENSG00000164305 | Inverse variance weighted | 7 | 1.04 (0.91, 1.20) | 0.54 |
|  |  | Simple mode | 7 | 0.58 (0.40, 0.83) | 0.03 |
|  |  | Weighted mode | 7 | 1.07 (0.97, 1.18) | 0.22 |
|  |  | MR Egger | 21 | 1.08 (0.85, 1.37) | 0.55 |
|  |  | Weighted median | 21 | 1.01 (0.91, 1.13) | 0.85 |
| EGFR | prot-a-909 | Inverse variance weighted | 21 | 0.96 (0.88, 1.05) | 0.37 |
|  |  | Simple mode | 21 | 1.08 (0.88, 1.32) | 0.48 |
|  |  | Weighted mode | 21 | 1.08 (0.89, 1.31) | 0.46 |
